# Sex Differences in Functional Gradients and Dynamic Functional Connectivity in Preschool‐Aged Children With ASD

**DOI:** 10.1111/cns.70562

**Published:** 2025-08-10

**Authors:** Guangrong Wu, Linfeng Song, Guomin Zhang, Yuanyuan Xu, Jie Fang, Siyan Xiong, Wei Yang, Lin Jiang

**Affiliations:** ^1^ Department of Radiology The Third Affiliated Hospital of Zunyi Medical University (The First People's Hospital of Zunyi) Zunyi Guizhou China

**Keywords:** autism spectrum disorder, brain networks, dynamic functional connectivity, gradient, machine learning models, male, preschool‐aged

## Abstract

**Background:**

The prevalence of autism spectrum disorder (ASD) is significantly higher in males than in females; although the underlying etiology remains unclear. This study aimed to investigate the multi‐scale reorganization of brain networks in preschool‐aged boys with ASD and their impact on clinical symptoms.

**Methods:**

A total of 54 children with ASD (40 boys and 14 girls) and 44 typically developing (TD) children (28 boys and 16 girls), aged between 2 and 6 years, were recruited for this study. Functional gradient analysis and dynamic functional connectivity were used to examine differences in the hierarchical organization of brain functional networks between preschool‐aged boys and girls with ASD compared to their corresponding typically developing peers. Subsequently, multiple machine learning models were applied to evaluate the classification performance of the identified abnormal features in distinguishing ASD from TD.

**Results:**

The results showed that the ASD group exhibited functional gradient abnormalities in multiple brain networks: (1) In boys with ASD, primary gradient abnormalities were identified in the dorsal attention network, limbic network, ventral attention network, and default mode network (DMN), whereas in girls with ASD, primary gradient abnormalities were only found in the DMN; (2) Secondary gradient abnormalities in boys with ASD were found in the sensorimotor network (SMN), ventral attention network, and DMN, while in girls with ASD, secondary gradient abnormalities were restricted to the DMN and SMN; (3) Third gradient abnormalities in boys with ASD were observed only in the visual network, whereas in girls with ASD, abnormalities were present in the limbic network, SMN, and visual network; (4) Enhanced dynamic functional connectivity was detected in boys with ASD only in state 1.

**Conclusion:**

Preschool‐aged boys and girls with ASD exhibit significant sex differences in functional gradients and dynamic functional connectivity, underscoring the complexity and heterogeneity of ASD. These findings provide a novel theoretical framework for understanding the neuroimaging mechanisms underlying ASD.

## Graphical abstract

This study investigates sex‐specific alterations in the functional organization of the brain in preschool‐aged children with ASD. Boys with ASD exhibited widespread abnormalities, primarily in the primary functional gradient. In contrast, girls showed more localized disruptions, predominantly in the third gradient. Additionally, increased dynamic functional connectivity was observed exclusively in boys, highlighting distinct neurodevelopmental trajectories. This flowchart illustrates the analysis pipeline for functional gradients and dynamic functional connectivity (dFC) in preschool children with autism spectrum disorder (ASD), comparing boys and girls with their respective typically developing (TD) control groups. Resting‐state fMRI data were preprocessed and used to extract time series. dFC and functional gradient analyses were then conducted independently. dFC analysis revealed significant group differences only among boys, with no differences observed between ASD and TD girls. In contrast, functional gradient analysis showed that group differences in boys were primarily localized to the primary gradient, while in girls, they were predominantly found in the third gradient.

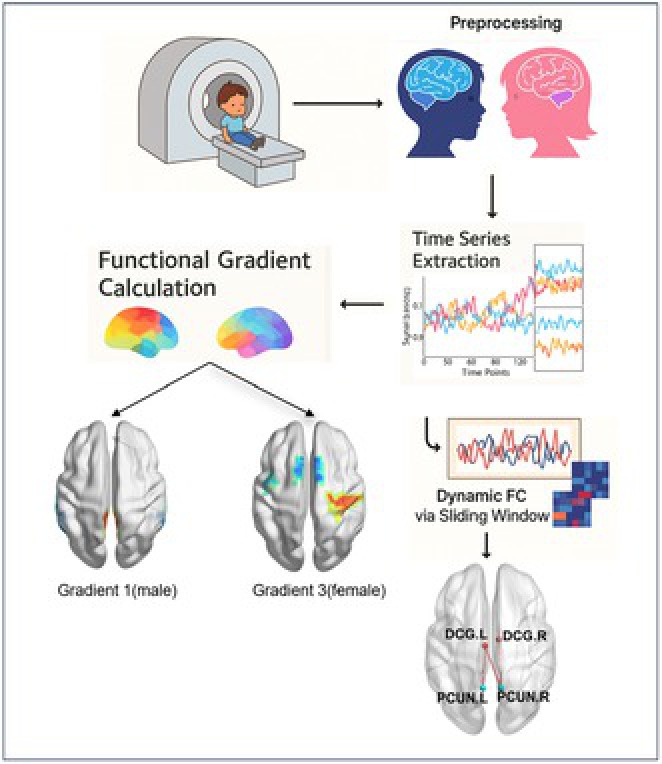

## 1 Introduction

Autism Spectrum Disorder (ASD) is a neurodevelopmental condition characterized by impairments in social communication and the presence of repetitive, stereotyped behaviors. Its pathophysiological mechanisms involve complex interactions among genetic, epigenetic, and environmental factors [1]. Globally, the prevalence of ASD has risen from 0.67% in 2000 to approximately 1.5% in recent years [2], with a pronounced male predominance (male‐to‐female ratio of 4:1) [3]. Theories such as the “female protective effect”—which proposes that females require a higher genetic load for symptom manifestation—and the “extreme male brain” theory attempt to account for this gender disparity, but the underlying neuroimaging mechanisms remain unclear [4]. ASD diagnosis is currently based solely on behavioral criteria outlined in the diagnostic and statistical manual of mental disorders, fifth edition, and lacks objective biological markers. Children under the age of six exhibit the highest levels of brain plasticity, and interventions during this critical window can significantly influence future cognitive and behavioral development [5]. Consequently, development of neuroimaging‐based early diagnostic tools for preschool‐aged children with ASD has become a major focus of international research.

The preschool age range (2–6 years) represents a critical developmental period characterized by rapid synaptic pruning, heightened plasticity, and large‐scale functional network reorganization. During this window, core brain networks—such as the default mode network (DMN), sensorimotor network (SMN), and ventral attention network (VAN)—undergo significant maturation, shifting from local to distributed connectivity patterns [6, 7]. Resting‐state fMRI (rs‐fMRI) studies in typically developing children during this stage have demonstrated a trajectory of increasing inter‐network integration and functional specialization. In contrast, ASD preschoolers show atypical connectivity signatures, such as hyperconnectivity in SMN and hypoconnectivity in DMN, which are thought to precede overt behavioral symptoms [8]. However, conducting rs‐fMRI in young children remains technically challenging due to high motion sensitivity, the need for sedation, and the absence of age‐specific brain templates, all of which may compromise spatial normalization and interpretation [9]. These challenges highlight both the importance and the complexity of early developmental brain research in ASD. Children under the age of six exhibit the highest levels of brain plasticity, and interventions during this critical window can significantly influence future cognitive and behavioral development [5]. Consequently, the development of neuroimaging‐based early diagnostic tools for preschool‐aged children with ASD has become a major focus of international research.

Resting‐state functional magnetic resonance imaging (rs‐fMRI) has offered a valuable approach for investigating the neural mechanisms underlying ASD. Conventional functional connectivity analyses have revealed a “dual pattern” in individuals with ASD, characterized by reduced intra‐network connectivity within the default mode network (DMN) and increased connectivity within the sensorimotor network [10]. Moreover, studies have identified notable alterations in dynamic functional connectivity (dFC) in ASD, such as heightened dFC between the right dorsolateral prefrontal cortex and the left precentral gyrus, which has been found to correlate with symptom severity [11].

However, these static measures are insufficient to capture the full heterogeneity of ASD symptoms. Emerging multiscale analysis methods, such as functional gradients and dFC, offer new avenues to address this limitation. Functional gradients employ dimensionality reduction techniques to map brain regions into low‐dimensional representational spaces, quantifying the hierarchical organization of functional networks from primary sensory areas to higher‐order association cortices [12]. Recent studies have shown that functional gradient changes in ASD are primarily associated with functional network alterations in adolescents and structural brain changes, including gray matter density and volume, in adults [8]. Furthermore, abnormal functional gradient patterns in ASD may affect higher‐order cognitive functions [13]. In contrast, dFC captures dynamic reorganization of brain networks using sliding time windows, revealing reduced state transition frequency and prolonged dwell time in specific states, such as default network‐dominated modes, termed “dynamic rigidity” in ASD patients [9]. Notably, functional gradient abnormalities may indicate structural disruptions in brain network hierarchy, while dFC abnormalities may reflect impaired temporal coordination. Together, these complementary approaches provide a comprehensive framework to elucidate multiscale functional dysregulation in ASD [14].

Despite significant advances in understanding the neural mechanisms of ASD, existing rs‐fMRI studies predominantly focus on school‐aged or adult ASD populations. However, the age range of 2 to 6 years represents a critical period of synaptic pruning and network differentiation [7], during which brain network abnormalities may serve as precursor markers for the consolidation of ASD symptoms [6]. While males constitute the majority of ASD patients, approximately 80% of imaging studies utilize mixed‐gender samples. Recent evidence suggests that female ASD patients may compensate for social deficits through enhanced prefrontal‐amygdala connectivity, indicating the presence of gender‐specific neural phenotypes [15]. Conducting an analysis of male samples is crucial for elucidating core pathological mechanisms. The integration of functional gradients and dFC remains in its infancy. Does the hierarchical organization of gradients constrain dynamic connectivity state transitions? How do these mechanisms collectively contribute to ASD behavioral phenotypes? These questions warrant urgent exploration.

Building on the aforementioned advancements, this study focuses on male children with ASD aged 2–6 years, integrating functional gradients and dFC methods for the first time. Additionally, machine learning models are employed to identify which abnormal features exhibit the highest discriminative power for ASD. We hypothesize that preschool‐aged boys with ASD exhibit concurrent abnormalities in the functional gradients and dynamic functional connectivity within specific brain networks, exhibiting more extensive abnormalities compared to preschool‐aged girls with ASD. Furthermore, we propose that certain abnormal features in ASD may demonstrate stronger discriminative ability in distinguishing between boys with ASD and typically developing (TD) boys.

## 2 Methods

### 2.1 Participants

This study recruited 54 children with ASD (40 boys and 14 girls), aged 2 to 6 years, who were evaluated and treated at the Pediatric Rehabilitation Department of the Third Affiliated Hospital of Zunyi Medical University between April 2022 and August 2024. The diagnosis of ASD was based on the criteria outlined in the diagnostic and statistical manual of mental disorders, fifth edition and the childhood autism rating scale (CARS) [16], with CARS scores exceeding 30. A total of 44 age‐ and sex‐matched TD children (28 boys and 16 girls) were recruited through advertisements. Informed written consent was obtained from the parents or guardians of all participants after they were fully informed about the potential risks and benefits of the study and assured of the confidentiality and privacy of their children's medical records. All participants were confirmed to be free of metal implants, brain abnormalities, drug dependencies, and other MRI contraindications. This study was approved by the Ethics Committee of the Third Affiliated Hospital of Zunyi Medical University (The First People's Hospital of Zunyi) (Ethics approval number: 2023‐1‐282).

### 2.2 Image Acquisition and Analyses

MRI examinations were performed with a 3.0‐Tesla unit (MAGNETOM VIDA, SIEMENS Healthcare, Germany) at the First People's Hospital of Zunyi. Initially, routine MRI scans (axial T1 weighted Imaging, axial T2 weighted Imaging, and T2 FLAIR) were performed to exclude participants with major structural brain abnormalities. The following settings were applied to obtain high‐resolution three‐dimensional T1‐weighted structural data: repetition time (TR) = 2300.0 ms, echo time (TE) = 2.98 ms, voxel = 1.0 × 1.0 × 1.1 mm, flip angle = 9°, field of view (FOV) = 256 mm × 256 mm, slice thickness = 1.10 mm, slices = 144. Resting‐state functional imaging data were collected using a whole‐brain gradient echo‐planar imaging sequence with the following parameters: TR = 2000.0 ms, TE = 28.0 ms, voxel = 3.0 × 3.0 × 3.0 mm, FOV = 240 mm × 240 mm, slices = 36, slice thickness = 3.0 mm. During scanning, participants lay in a supine position, with their whole body while lying as still as possible. Foam pads were used to stabilize the head and restrict movement, and soft earplugs were provided to reduce noise interference. Participants received a combination of chloral hydrate and syrup (40 mg/kg) orally, with a maximum single dose of 1 g. Once participants fell asleep, their level of consciousness was assessed. If they showed no response to mild pain stimuli, they were placed on the examination table in a supine position. If a participant awoke and could not resume the scan, they were withdrawn from the study.

### 2.3 Preprocessing

The T1WI‐3D‐MP RAGE and blood oxygen level–dependent (BOLD) images were preprocessed using the MATLAB‐based software DPABI V6.1 (http://rfmri.org/dpabi). The preprocessing steps included the following eight procedures: Convert the raw DICOM data into NIFTI format; Remove the first 10 time points of the rs‐fMRI data; Perform temporal slice correction on the 200 time points of the BOLD data to align the same brain regions to the same time point for scanning; Participants exhibiting head movement greater than 2 mm in the *x*, *y*, or *z* directions and/or rotation angles exceeding 2° were excluded from further analysis. Frame‐wise displacement was calculated for each volume to quantify micro head motion. Volumes with frame‐wise displacement greater than 0.5 mm were identified as contaminated by micro head motion and were excluded from the analysis. To further minimize the impact of micro head motion, a scrubbing procedure was applied, and nuisance regressors for six motion parameters (translation and rotation) and their first‐order derivatives were incorporated into the general linear model. Remove non‐brain tissues such as scalp and skull from the images, retaining 90% of the brain region to improve spatial registration accuracy and limit the analysis scope; Register the BOLD functional images to the high‐resolution T1‐weighted images and spatially normalize them to the Montreal Neurological Institute space; Remove noise contributions from cerebrospinal fluid and other sources; Remove linear trends to eliminate low‐frequency drifts.

### 2.4 Functional Gradient

First, we extracted voxel‐level BOLD time series from all seven brain networks as defined by the Yeo template [17]. Additionally, we divided the cortical surface into 400 regions and extracted the average BOLD time series for each region. For each participant, we computed seven brain network functional connectivity matrices. These matrices were calculated by determining Pearson correlation coefficients between the BOLD time series of each voxel within a brain network and the average time series of each cortical region. Fisher's r‐to‐z transformation was applied to normalize the functional connectivity matrices. To align with previous studies [18, 19, 20, 21], we constructed a cosine similarity matrix for each brain network by selecting the top 10% of voxel‐wise connectivity values. This resulted in a similarity matrix for each participant, capturing the spatial patterns of microstructural organization.

Functional gradients for the seven brain networks were computed using the BrainSpace toolbox (http://github.com/MICA‐MNI/BrainSpace). The similarity matrices were projected onto a low‐dimensional manifold using diffusion map embedding. This manifold learning algorithm identified spatial axes of connectivity variation across different voxels and was used to extract gradient components ordered by descending eigenvalues in the similarity matrix [22]. We set the alpha parameter of the gradient algorithm to 0.5 (alpha = 0 for maximum influence; alpha = 1 for minimum influence) and the *t* parameter to 0, consistent with previous studies [23, 24]. A group‐level gradient template was created based on the average functional connectivity matrix from all participants, including both baseline and follow‐up data. Individual‐level gradients were estimated and aligned to the template using Procrustes alignment. Finally, the individual gradient maps were smoothed (6 mm full‐width at half‐maximum Gaussian kernel) and normalized to *Z*‐scores [25]. To reduce spatial noise and enhance the signal‐to‐noise ratio, a 6 mm full‐width at half‐maximum (FWHM) Gaussian smoothing kernel was applied. The selection of this kernel size balances spatial specificity and sensitivity: larger kernels (e.g., 8–10 mm) can enhance statistical power but reduce anatomical precision, while smaller kernels (e.g., 4 mm) retain fine‐grained structure but may hinder the detection of distributed network effects. The 6 mm kernel is commonly adopted in resting‐state fMRI literature as a robust and practical standard (Spatial Smoothing Effect on Group‐Level Functional Connectivity during Resting and Task‐Based fMRI. Sens). We focused on the primary gradient components that explained the most variance and exhibited the highest interpretability, referred to as functional gradients in this study.

### 2.5 Dynamic Functional Connectivity

The dFC was analyzed using a sliding window approach implemented in the DynamicBC toolbox (http://www.restfmri.net/forum/DynamicBC). A window length of 50 TR and a step size of 1 TR were selected, consistent with commonly adopted practices in previous dFC studies involving clinical populations. For example, studies by Li et al. on sleep‐related disorders have demonstrated that this configuration offers good feasibility and stability in capturing meaningful brain dynamics [26, 27]. A window of this length provides a practical trade‐off between capturing temporal dynamics and ensuring stable correlation estimation in resting‐state data [28, 29, 30]. Similarly, a 1 TR step size enables high‐resolution tracking of transient brain state transitions without excessive redundancy [31]. While alternative parameters (e.g., 30 or 80 TR windows) can yield minor quantitative changes, prior studies have demonstrated that dFC state profiles and group comparisons remain qualitatively consistent across a range of reasonable parameter choices [32]. This process resulted in 151 sliding windows. Within each window, voxel‐wise Pearson correlation coefficients were computed, followed by Fisher *Z*‐transformation to improve normality. The standard deviation of *Z*‐values across windows was calculated to quantify the temporal variability of functional connectivity for each voxel. Based on this configuration, this process generated 151 windows. Within each sliding window, Pearson correlation coefficients were computed between all voxel time series. Consequently, each participant obtained a set of sliding window correlation maps. To enhance the normality of the correlation distribution, Fisher *Z*‐transformation was applied to convert the correlation maps into *Z*‐values. To assess the variability of dFC, the standard deviation of *Z*‐values was calculated for each voxel, reflecting the variance of the time series correlation coefficients. To further characterize the dynamic connectivity patterns, *k*‐means clustering was applied to group the dFC matrices into discrete recurrent states. To determine the optimal number of clusters, we examined the sum of squared errors (SSE) for *k* values ranging from 2 to 8 and plotted the SSE curve. The analysis showed that when *k* = 4, the SSE exhibited an “elbow point,” indicating a balance between model complexity and variance explanation. Additionally, we calculated the Silhouette coefficient and the Calinski‐Harabasz index to validate the clustering stability. Both metrics suggested that *k* = 4 provided the most robust separation between states while maintaining intra‐cluster cohesion. Therefore, we selected *k* = 4 for subsequent analyses.

### 2.6 Machine Learning Models

Multiple machine learning methods were employed to construct classification models for assessing the discriminative power of brain regions exhibiting functional gradient and dynamic functional connectivity abnormalities [33, 34]. First, the dataset was preprocessed by removing outliers and redundant features. The Pearson correlation was used to identify features with significant correlation with the target variable, thereby constructing a high‐quality input feature set. To ensure model performance stability and generalizability, leave‐one‐out cross‐validation (LOOCV) was employed for training and testing. In addition to the original LOOCV, nested cross‐validation (internally nested 5‐fold cross validation) was conducted to find the optimal parameters and prevent information leakage from the test set to avoid overfitting. In each iteration, one sample was used as the test set and the remaining samples as the training set to mitigate overfitting caused by the small sample size.

Three commonly used supervised learning models were applied to perform classification: (1) linear SVM: A linear kernel constructs a hyperplane that maximizes the margin between classes, with optimal parameters determined through grid search; (2) RBF SVM: A radial basis function kernel maps the data into a higher‐dimensional space to handle complex decision boundaries, with optimal parameters (*C* and *γ*) tuned through cross‐validation; (3) Random forest: An ensemble model based on multiple decision trees using bagging, with parameters such as the maximum number of features and the number of trees being optimized. The hyperparameters of Linear SVM are set to *C* = [1e‐3, 1e‐2, 1e‐1, 10]; Gaussian Kernel SVM (RBFSVM) hyperparameters are set to *C* = [1e‐3, 1e‐2, 1e‐1, 1, 10], gamma = [1e‐3, 1e‐2, 1e‐1, 1, 10]; The hyperparameters of the random forest are set to optimize the number of trees = {50, 100, 200}, maximum splitting depth = {5, 10, 20, 30, ∞}, and minimum leaf node sample size = {1, 2, 4}.

Model performance was evaluated using a confusion matrix, which quantified the classification results in terms of true positive, true negative, false positive, and false negative values. Classification performance metrics, including accuracy, specificity, sensitivity, ROC curve with 95% confidence interval, and AUC, were calculated to evaluate model performance. Using these methods, the classification ability of the three models under different feature combinations was assessed, clarifying the importance and predictive contribution of each feature in the models. All code and intermediate outputs related to preprocessing, feature selection, and model evaluation are available on GitHub [https://github.com/nemowgr/Machine‐learning‐models], and key results (e.g., feature lists, confusion matrices, cross‐validation splits) are included as Tables S4–S6.

### 2.7 Statistical Analysis

Normality tests for continuous data were conducted using the Shapiro–Wilk test. Differences in age between ASD and TD were analyzed using the Mann–Whitney *U* test in SPSS 29.0.

Two‐sample *t*‐tests were used to compare differences in functional gradients and dynamic functional connectivity between ASD patients and TD, with age and mean framewise displacement (FD) included as covariates Multiple comparisons for functional gradients were corrected using Gaussian random field (GRF) theory, with a cluster significance threshold of *p* < 0.05 and a voxel significance threshold of *p* < 0.05. Multiple comparisons for dynamic functional connectivity were corrected using the false discovery rate (FDR), with a significance threshold of *p* < 0.05. Pearson's correlation analysis was used to examine the relationship between differences in functional gradients and dynamic functional connectivity, clinical variables, and age. The significance level was set at *p* < 0.05 (two‐sided).

## 3 Results

### 3.1 Demographic and Clinical Data

There were no significant age differences between the ASD and TD groups in males, or between the ASD and TD groups in females (Table 1). Their CARS scores and disease duration are shown in Table 1 (*p* > 0.05).

**TABLE 1:** Demographic characteristics of the sample.

		ASD	TD	*U*	*p*
Male	Number of subjects	40	28	—	—
	Age (months)	44.62 ± 14.16	49.5 [37.5, 65.5]	418	0.078^a^
	CARS	37.35 ± 4.41	—	—	—
	Duration (months)	17.73 ± 9.88	—	—	—
Female	Number of subjects	14	16	—	—
	Age (months)	45.21 ± 10.72	50.5 [38.0, 66.5]	83.5	0.244^a^
	CARS	36.5 [35.0, 40.75]	—	—	—
	Duration (months)	20.07 ± 9.18	—	—	—

Abbreviations: CARS, childhood autism rating scale; TD, typically developing.

^a^ Mann–Whitney *U* test; *U* = Mann–Whitney *U* nonparametric test statistic.

### 3.2 Functional Gradient

#### 3.2.1 Functional Gradient of the Dorsal Attention, Limbic, Somatomotor and Visual in Males

**FIGURE 1:**
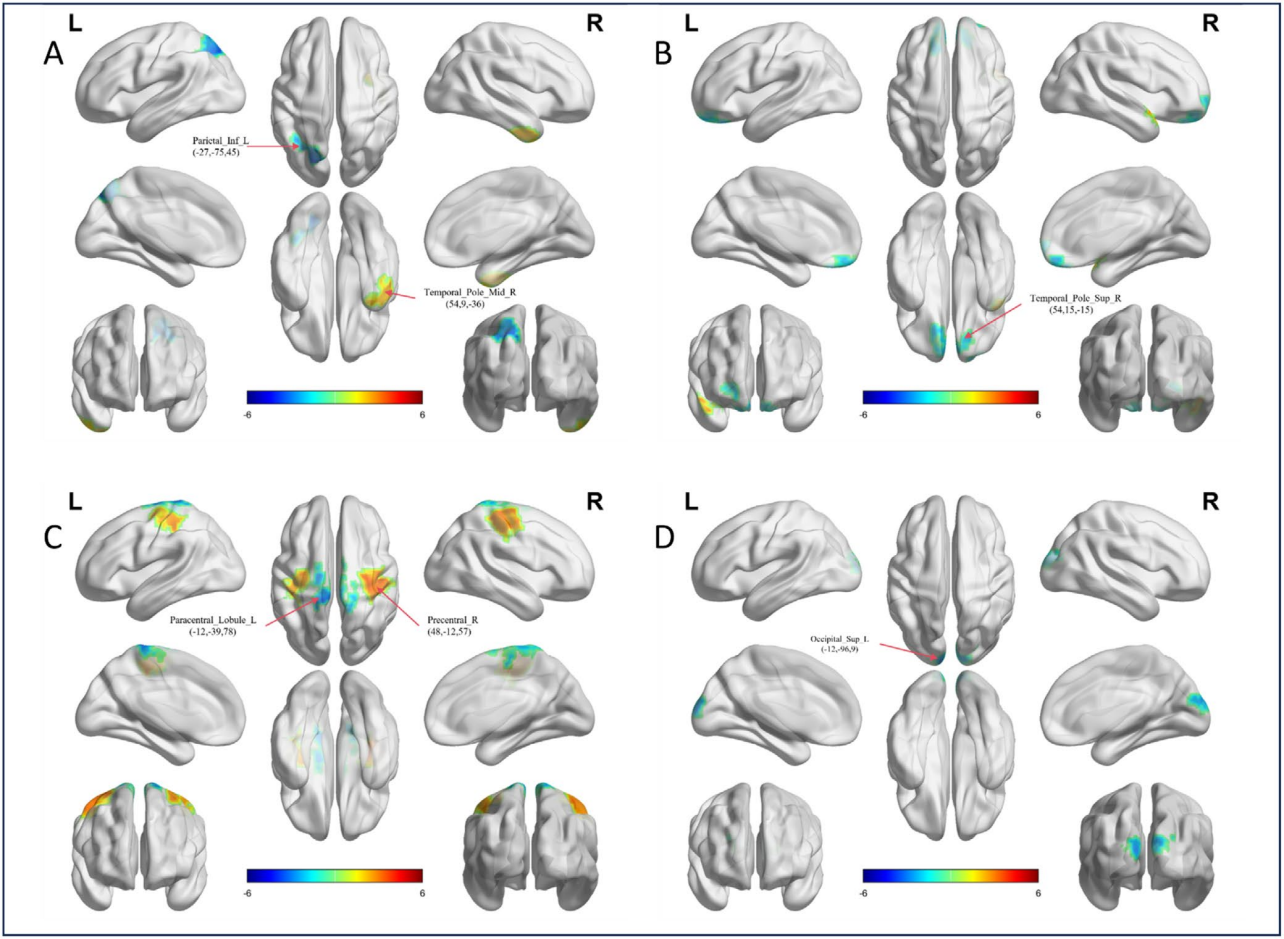
The functional gradient differences in brain regions of the dorsal attention network (DAN), limbic network (LIM), somatomotor network (SMN), and visual network (VIS) in males. (A) The left inferior parietal, but supramarginal and angular gyri and right temporal pole (middle temporal gyrus) in the DAN show significant group‐level differences in gradient 1 between ASD and TD groups. Surface rendering was generated using BrainNet Viewer. (B) The right temporal pole (superior temporal gyrus) in the LIM shows a difference in gradient 1 between the two groups. (C) The left paracentral lobule and right precentral gyrus in the SMN show a difference in gradient 2 between the two groups. (D) The left superior occipital gyrus in the VIS shows a difference in gradient 3 between the two groups.

In the present study, preschool male children with ASD exhibited abnormal functional gradient patterns in the dorsal attention network (DAN), limbic network (LIM), somatomotor network (SMN), and visual network (VIS) (Table 2). We extracted the first to third functional gradients for the seven brain networks in ASD patients and found significant intergroup differences in the first gradient of the DAN, in the left inferior parietal lobule—including the supramarginal and angular gyri—and in the right temporal pole (middle temporal gyrus) (Figure 1A). Compared to the TD group, individuals with ASD showed lower functional gradient scores in the left inferior parietal lobule—including the supramarginal and angular gyri—and higher scores in the right temporal pole (middle temporal gyrus). Similarly, in the LIM, only the first gradient exhibited significant intergroup differences, with increased functional gradient scores in the right temporal pole (superior temporal gyrus) (Figure 1B) in the ASD group compared to the TD group. For the SMN, significant differences were observed in the second gradient at the left paracentral gyrus and right precentral gyrus (Figure 1C), with reduced scores in the left paracentral gyrus and increased scores in the right precentral gyrus in the ASD group. In the VIS, significant differences were found in the left superior occipital gyrus (Figure 1D), with reduced functional gradient scores in the ASD group compared to the TD group (Two‐sample *t*‐tests, *p* < 0.05, GRF corrected, two‐tailed).

**TABLE 2:** Functional gradient of the DAN, LIM, SMN and VIS in males.

		Brain regions (AAL)	Voxels, n	MNI coordinates, mm (*x*, *y*, *z*)			Peak *t* values
DAN	Gradient 1	Parietal_Inf_L	306	−27	−75	45	−4.1378
		Temporal_Pole_Mid_R	223	54	9	−36	3.3581
LIM	Gradient 1	Temporal_Pole_Sup_R	225	54	15	−15	3.4848
SMN	Gradient 2	Paracentral_Lobule_L	1062	−12	−39	78	−4.7304
		Precentral_R	615	48	−12	57	4.1554
VIS	Gradient 3	Occipital_Sup_L	232	−12	−96	9	−3.4077

*Note:* GRF‐corrected (voxel *p* value < 0.001 and cluster *p* value < 0.05).

Abbreviations: AAL, automated anatomical labeling; DAN, dorsal attention network; GRF, Gaussian random field; LIM, limbic network; MNI, Montreal neurological institute; Occipital_Sup_L, left superior occipital gyrus; Paracentral_Lobule_L, left paracentral lobule; Parietal_Inf_L, left inferior parietal, but supramarginal and angular gyri; Precentral_R, right precentral gyrus; SMN, somatomotor network; Temporal_Pole_Mid_R, right temporal pole (middle temporal gyrus); Temporal_Pole_Sup_R, right temporal pole (superior temporal gyrus); VIS, visual network.

#### 3.2.2 Functional Gradient of the VAN and DMN in Males

**FIGURE 2:**
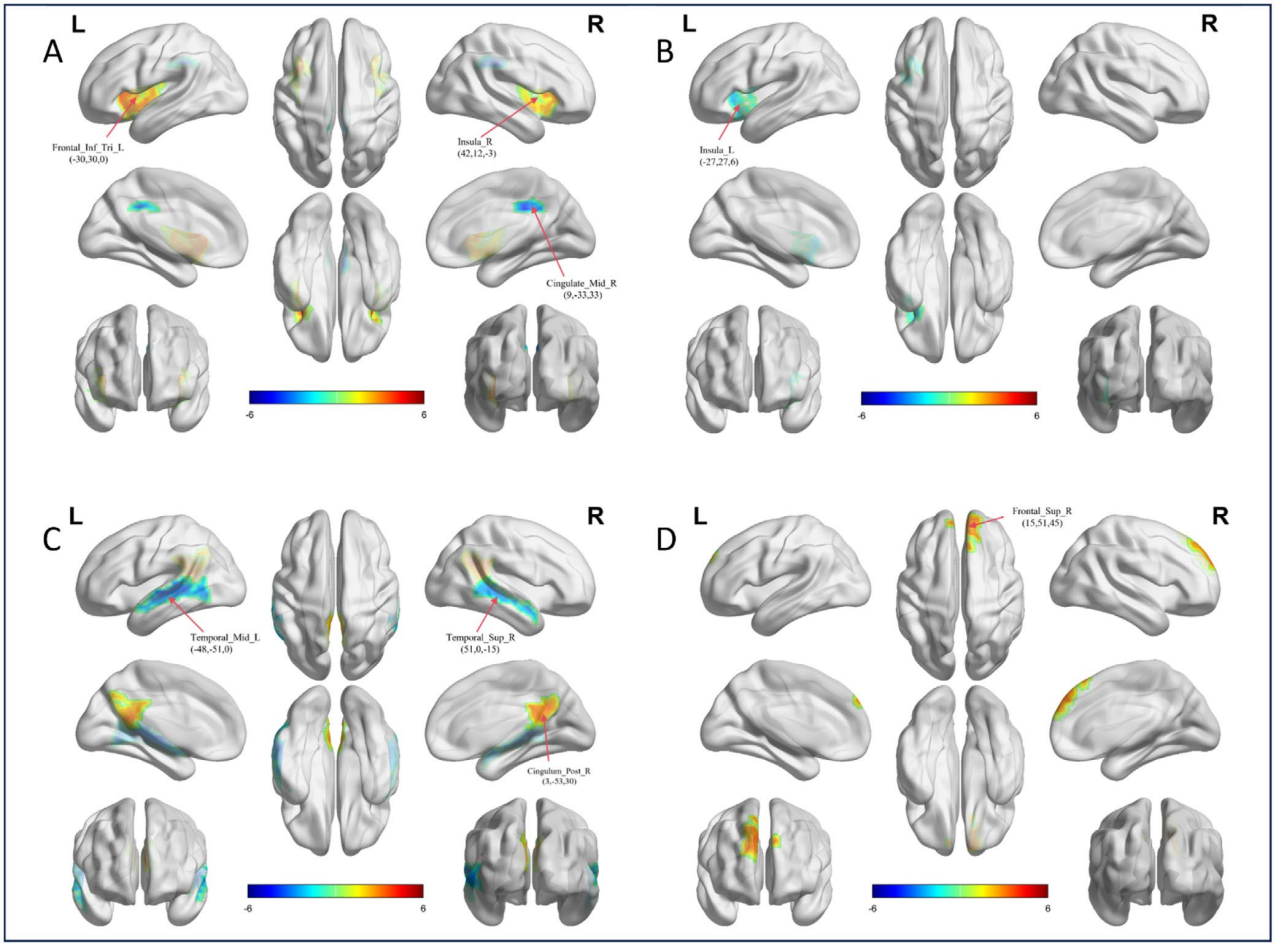
The functional gradient differences in brain regions of the ventral attention network (VAN) and default mode network (DMN) in males. (A) The left inferior frontal gyrus (triangular part), right insula, and right middle cingulate gyrus in the VAN show significant group‐level differences in gradient 1 between the ASD and TD groups. Surface rendering was generated using BrainNet Viewer. (B) The left insula in the VAN shows a difference in gradient 2 between the two groups. (C) The left middle temporal gyrus, right posterior cingulate gyrus, and right superior temporal gyrus in the DMN show differences in gradient 1 between the two groups. (D) The right superior frontal gyrus in the DMN shows a difference in gradient 2 between the two groups.

The ventral attention network (VAN) and DMN functional gradient results revealed significant group‐level differences in both the primary and secondary gradients between the ASD and TD groups (Table 3). In the VAN network, significant differences were observed in the primary gradient at the left inferior frontal gyrus (triangular part) and right insula, as well as in the secondary gradient at the left insula (Figure 2A,B). Specifically, the functional gradient scores for the left inferior frontal gyrus (triangular part) and right insula were increased in the ASD group compared to the TD group. Conversely, the functional gradient scores for the right middle cingulate gyrus and the left insula were decreased in the ASD group compared to the TD group. In the DMN, significant differences were observed in the primary gradient at the left middle temporal gyrus, right posterior cingulate gyrus, and right superior temporal gyrus (Figure 2C). Additionally, significant differences were found in the secondary gradient at the right superior frontal gyrus (Figure 2D). Notably, in the ASD group, the functional gradient scores for the right posterior cingulate gyrus and right superior frontal gyrus were increased, while the scores for the left middle temporal gyrus and right superior frontal gyrus were decreased compared to the TD group (Two‐sample *t*‐tests, *p* < 0.05, GRF corrected, two‐tailed).

**TABLE 3:** Functional gradient of the VAN and DMN in males.

		Brain regions (AAL)	Voxels, *n*	MNI coordinates, mm (*x*, *y*, *z*)			Peak *t* values
VAN	Gradient 1	Frontal_Inf_Tri_L	386	−30	30	0	4.0334
		Insula_R	329	42	12	−3	3.4225
		Cingulate_Mid_R	212	9	−33	33	−4.8423
	Gradient 2	Insula_L	192	−27	27	6	−2.8382
DMN	Gradient 1	Temporal_Mid_L	682	−48	−51	0	−4.2004
		Cingulum_Post_R	537	3	−54	30	3.9370
		Temporal_Sup_R	445	51	0	−15	−3.4780
	Gradient 2	Frontal_Sup_R	397	15	51	45	4.0357

*Note:* GRF‐corrected (voxel *p* value < 0.001 and cluster *p* value < 0.05).

Abbreviations: AAL, automated anatomical labeling; Cingulate_Mid_R, right middle cingulate gyrus; Cingulum_Post_R, right posterior cingulate gyrus; DMN, default mode network; Frontal_Inf_Tri_L, left inferior frontal gyrus (triangular part); Frontal_Sup_R, right superior frontal gyrus; GRF, Gaussian random field; Insula_L, left insula; Insula_R, right insula; MNI, Montreal neurological institute; Temporal_Mid_L, left middle temporal gyrus; Temporal_Sup_R, right superior temporal gyrus; VAN, ventral attention network.

#### 3.2.3 Functional Gradient of the DMN, LIM, SMN and VIS in Females

**FIGURE 3:**
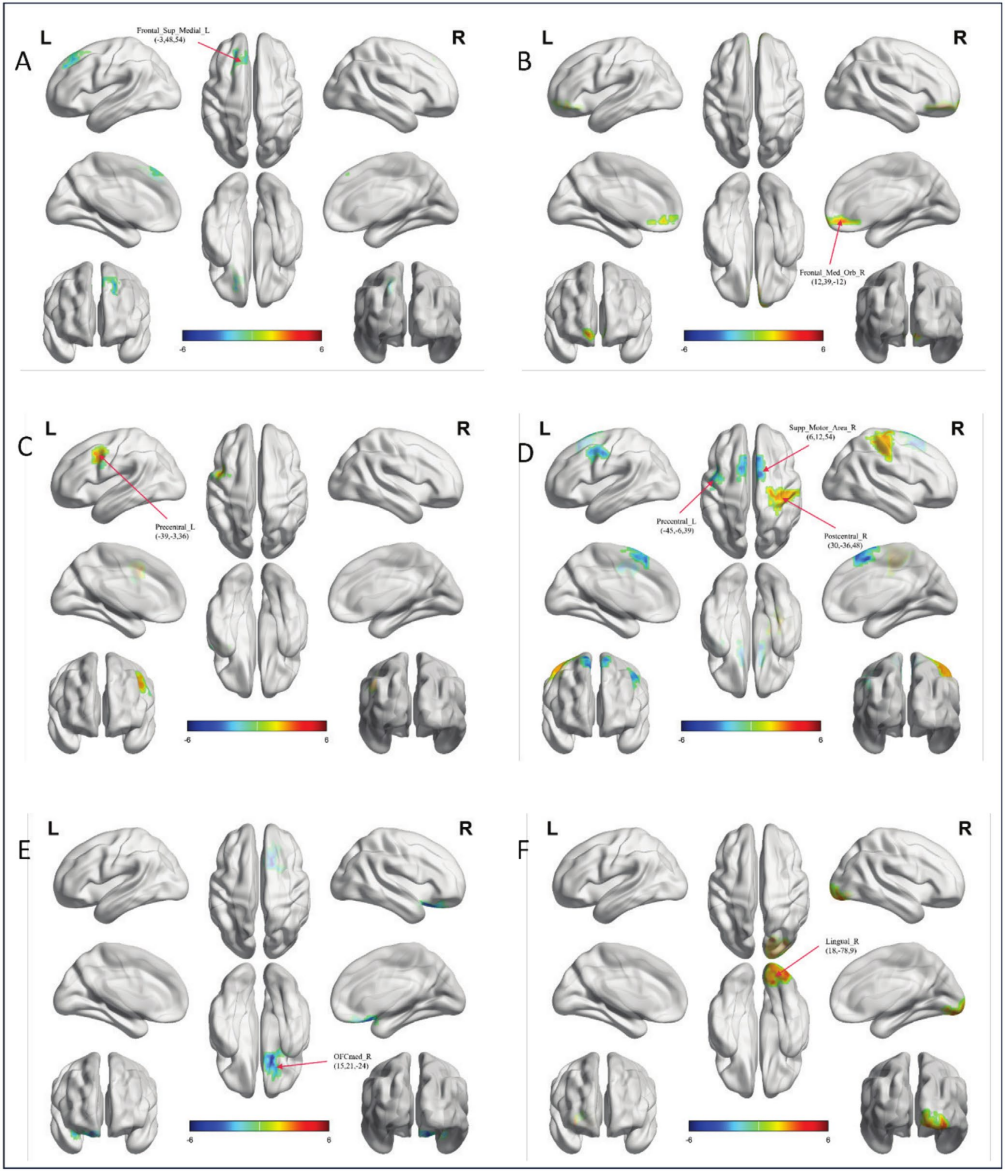
Functional gradient of the DMN, LIM, SMN and VIS in females. (A) The left superior frontal gyrus in the DMN show significant group‐level differences in gradient 1 between the ASD and TD groups. Surface rendering was generated using BrainNet Viewer. (B) The right superior frontal gyrus (medial orbital) in the DMN shows a difference in gradient 2 between the two groups. (C) The left precentral gyrus in the SMN show differences in gradient 2 between the two groups. (D) The right postcentral gyrus, right supplementary motor area and left precentral gyrus in the SMN shows a difference in gradient 3 between the two groups; (E) The right medial orbital gyrus in the LIM show differences in gradient 3 between the two groups; (F) The right lingual gyrus in the VIS show differences in gradient 3 between the two groups.

Female children with ASD exhibit milder alterations in functional gradients compared to their male counterparts (Table 4). The key findings include increased gradient scores in the left precentral gyrus (second gradient of the SMN) (Figure 3C), the right postcentral gyrus (third gradient of the SMN) (Figure 3D), the right superior frontal gyrus (medial orbital; second gradient of the DMN) (Figure 3B), and the right lingual gyrus (third gradient of the VIS) (Figure 3F). Conversely, decreased gradient scores are observed in the left superior frontal gyrus (medial; primary gradient of the DMN) (Figure 3A), the right medial orbital gyrus (third gradient of the LIM) (Figure 3E), and the right supplementary motor area and left precentral gyrus (third gradient of the SMN) (Figure 3D) (Two‐sample *t*‐tests, *p* < 0.05, GRF corrected, two‐tailed).

**TABLE 4:** Functional gradient of the DMN, LIM, SMN, and VIS in females.

		Brain regions (AAL)	Voxels, *n*	MNI coordinates, mm (*x*, *y*, *z*)			Peak *t* values
DMN	Gradient 1	Frontal_Sup_Medial_L	194	−3	48	54	−4.8404
	Gradient 2	Frontal_Med_Orb_R	148	12	39	−12	3.9815
SMN	Gradient 2	Precentral_L	235	−39	−3	36	3.7027
	Gradient3	Postcentral_R	572	30	−36	48	3.9441
		Supp_Motor_Area_R	409	6	12	54	−4.3736
		Precentral_L	219	−45	−6	39	−3.9239
LIM	Gradient 3	OFCmed_R	132	15	21	−24	−5.0601
VIS	Gradient 3	Lingual_R	308	18	−78	9	3.7039

*Note:* GRF‐corrected (voxel *p* value < 0.001 and cluster *p* value < 0.05).

Abbreviations: AAL, automated anatomical labeling; DMN, default mode network; Frontal_Med_Orb_R, right superior frontal gyrus (medial orbital); Frontal_Sup_Medial_L, left superior frontal gyrus (medial); GRF, Gaussian random field; LIM, limbic network; Lingual_R, right lingual gyrus; MNI, Montreal neurological institute; OFCmed_R, right medial orbital gyrus; Postcentral_R, right postcentral gyrus; Precentral_L, left precentral gyrus; SMN, somatomotor network; Supp_Motor_Area_R, right supplementary motor area; VIS, visual network.

### 3.3 dFC

#### 3.3.1 Dynamic Functional Connectivity Clustering

**FIGURE 4:**
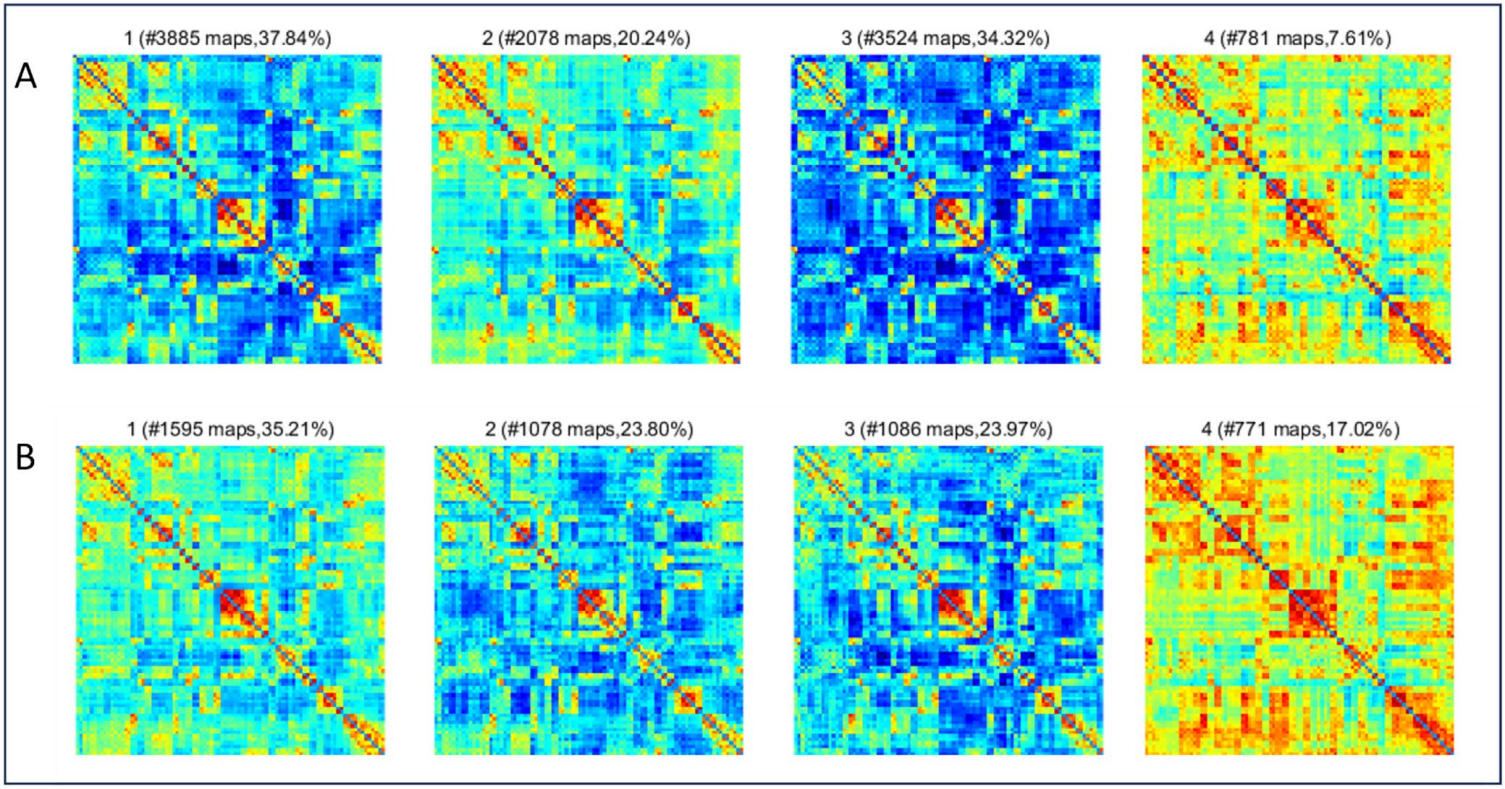
Brain feature clustering at *k* = 4, (A) the four cluster centroids for all subjects, with the frequency and percentage of each state displayed in males. (B) The four cluster centroids for all subjects, with the frequency and percentage of each state displayed in females.

**FIGURE 5:**
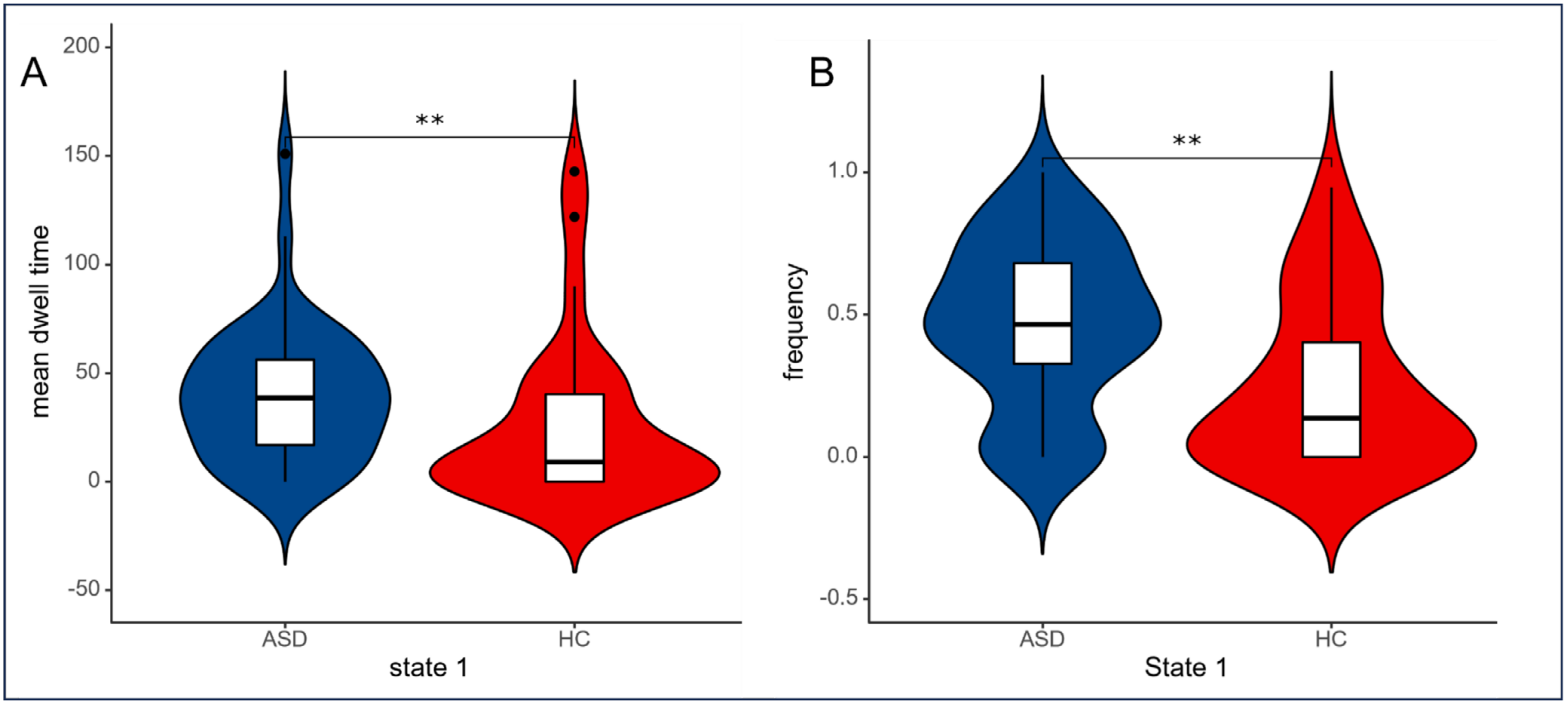
Between‐group comparison of time metrics derived from state transition vectors in males. ASD = autism spectrum disorder; TD = typically developing. An asterisk (*) in the bar chart indicates a significant statistical difference between the two groups. (A) The significant differences in the mean dwell time between groups in state 1; (B) The significant differences in the state transition frequency between groups in state 1.

**FIGURE 6:**
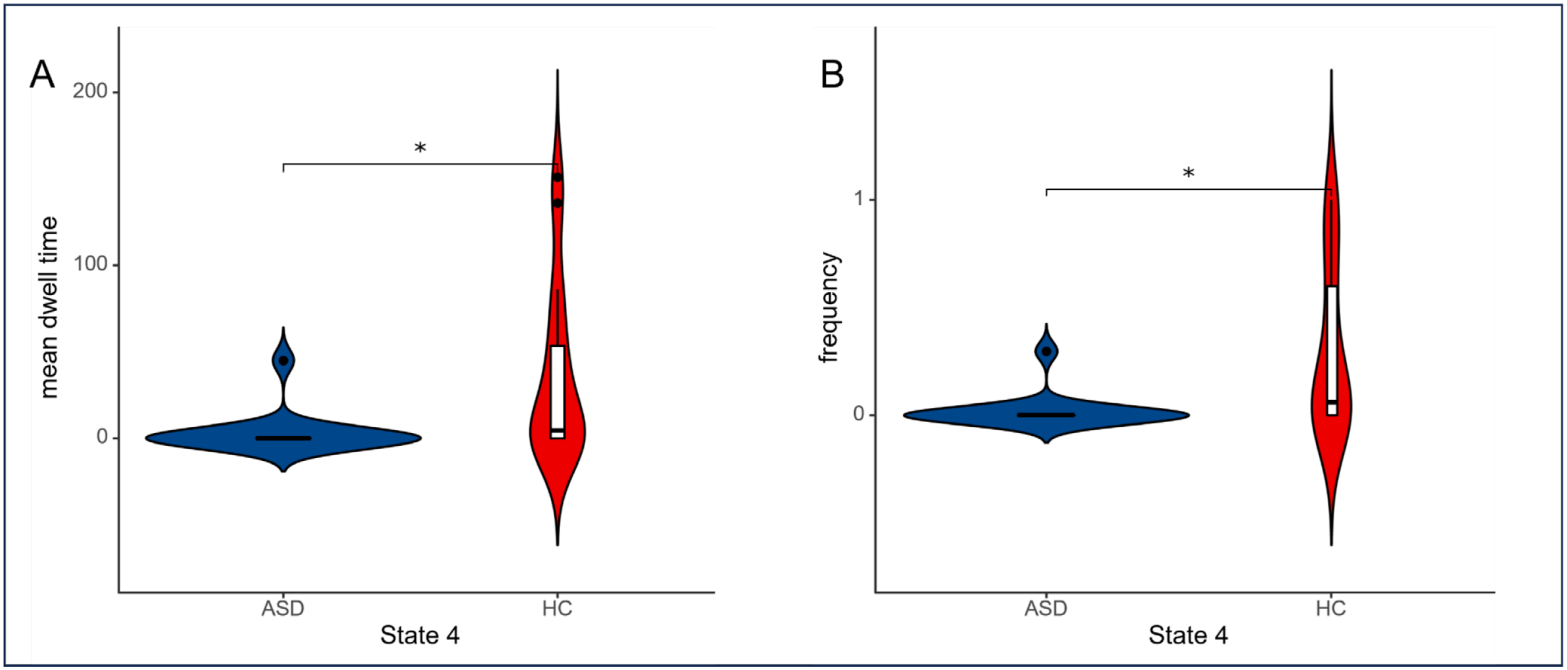
Between‐group comparison of time metrics derived from state transition vectors in females. ASD = autism spectrum disorder; TD = typically developing. An asterisk (*) in the bar chart indicates a significant statistical difference between the two groups. (A) The significant differences in the mean dwell time between groups in state 4; (B) The significant differences in state transition frequency between groups in state 4.

The K‐means clustering algorithm was applied to identify four distinct connectivity states in both male and female participants. In males, state 1 accounted for 37.84%, state 2 for 20.24%, state 3 for 34.32%, and state 4 for 7.61% (Figure 4A). Similarly, in females, state 1 accounted for 35.21%, state 2 for 23.80%, state 3 for 23.97%, and state 4 for 17.02% (Figure 4B). Comparisons of the temporal metrics for state transition vectors between the ASD and TD groups revealed that in males, the mean dwell time and state transition frequency from state 2 to state 4 showed no significant differences. In females, the mean dwell time and state transition frequency from state 1 to state 3 also showed no significant differences. However, ASD participants exhibited significantly increased mean dwell time (Figure 5A) and state transition frequency (Figure 5B) in state 1 compared to the TD group in males, while females showed a significant reduction in mean dwell time (Figure 6A) and state transition frequency (Figure 6B) for state 4 (Two‐sample *t*‐tests, *p* < 0.05, FDR corrected, two‐tailed).

#### 3.3.2 Clustering Analysis and Functional Connectivity Strength in Dynamic States

**FIGURE 7:**
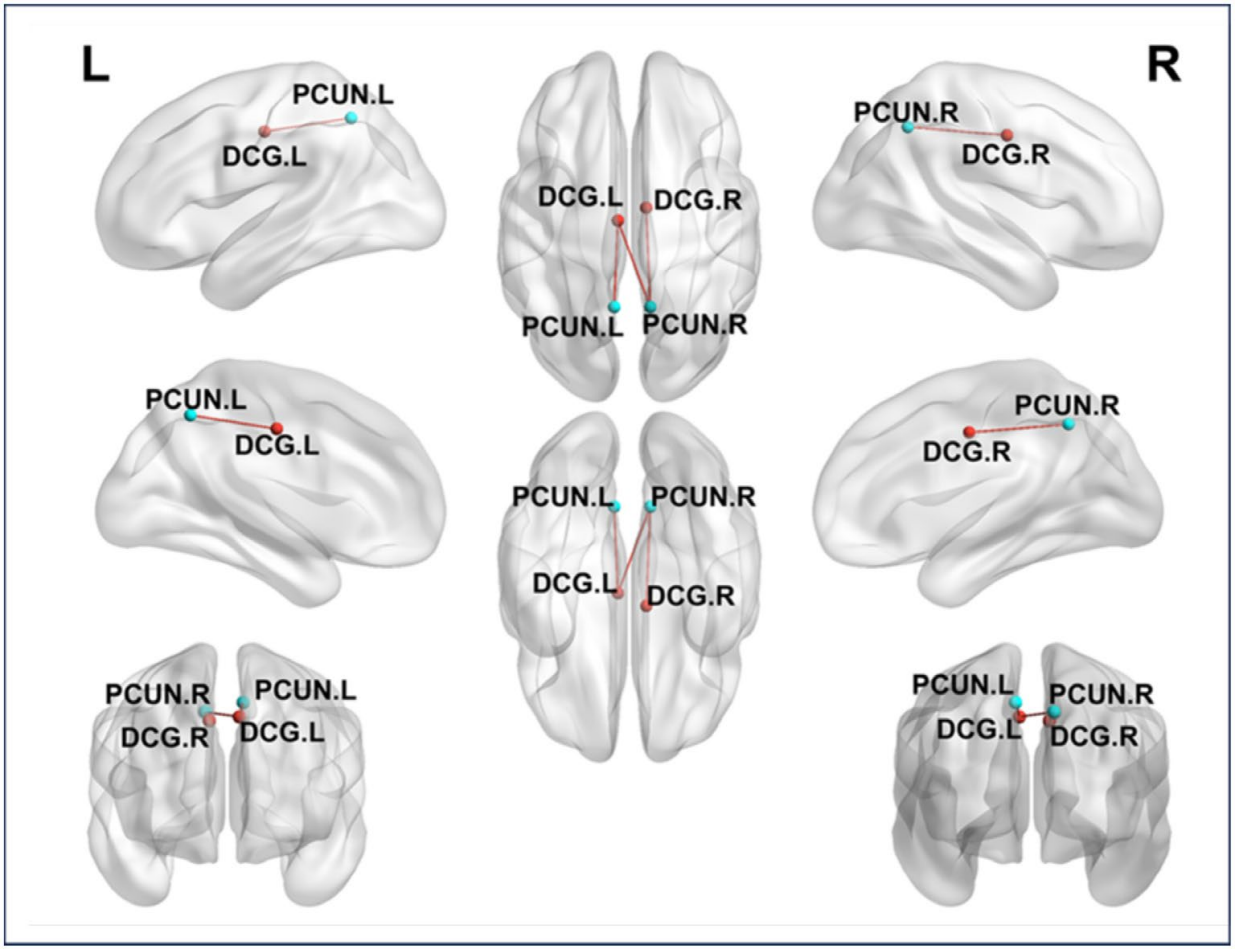
Significant connectivity changes in State 1 between ASD patients and TD; The red connections represent increased functional connectivity.

**FIGURE 8:**
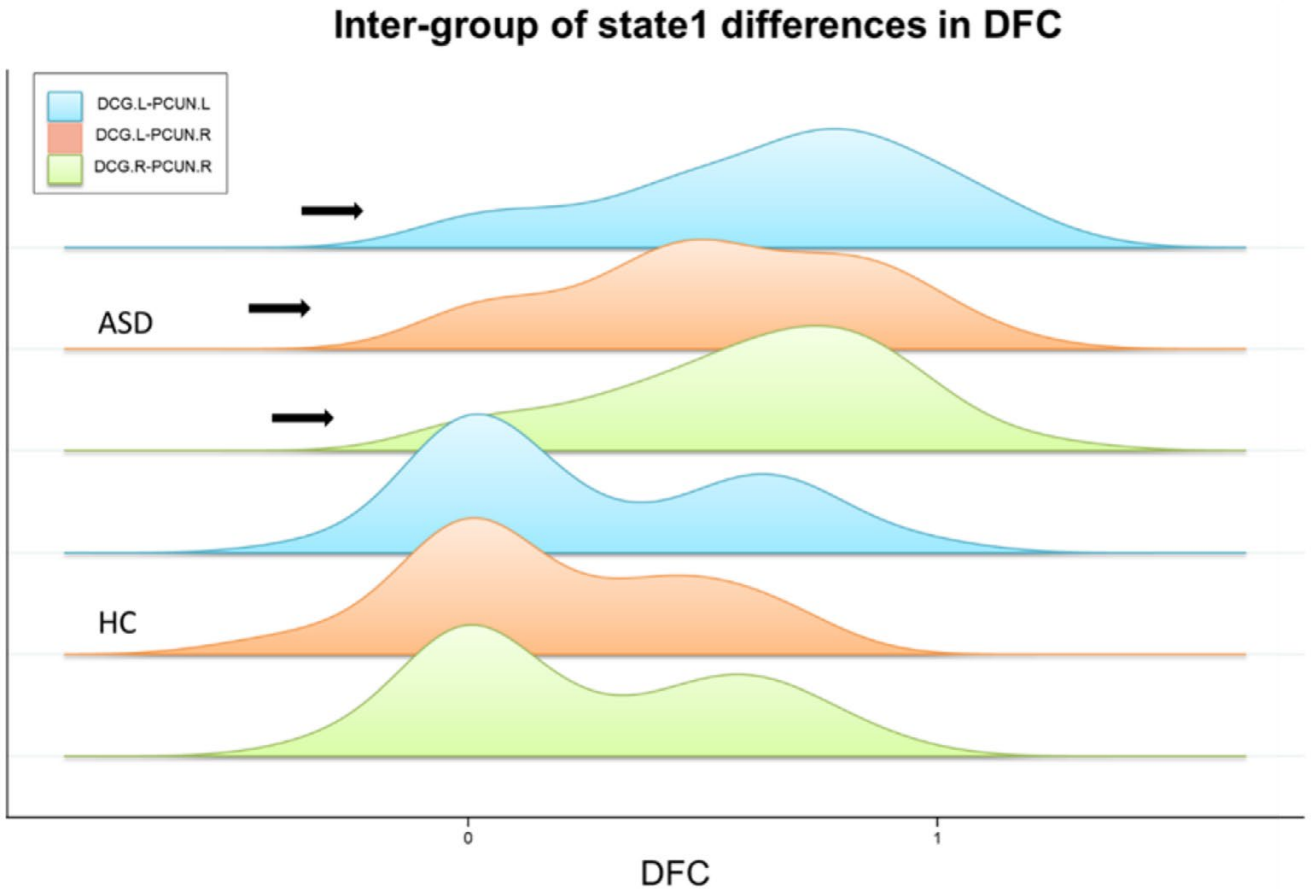
Histogram of between‐group differences in state 1 DFC. The direction of the significant differences between the ASD and TD. ASD, autism spectrum disorder; TD, typically developing; DCG.L‐PCUN.L, the dynamic functional connectivity between the left median cingulate and paracingulate gyri with left precuneus gyrus; DCG.L‐PCUN.R, the dynamic functional connectivity between left median cingulate and paracingulate gyri with right precuneus gyrus; DCG.R‐PCUN.R, the dynamic functional connectivity between right median cingulate and paracingulate gyri with right precuneus gyrus.

Two‐sample *t*‐tests were conducted to compare the intergroup differences in dFC strength between the ASD and TD groups across all states for both males and females. No statistically significant differences were found in states 2 to 4 among males and in all states among females; therefore, only the functional connectivity results of state 1 for males are reported here. Compared with the TD group, the ASD group showed increased connectivity in state 1 between the following brain regions: left median cingulate and paracingulate gyri with left precuneus gyrus, left median cingulate and paracingulate gyri with right precuneus gyrus, and right median cingulate and paracingulate gyri with right precuneus gyrus (Figures 7 and 8) (Two‐sample *t*‐tests, *p* < 0.05, FDR corrected, two‐tailed).

#### 3.3.3 Predictive Discrimination Performance of Various Machine Learning Models

**FIGURE 9:**
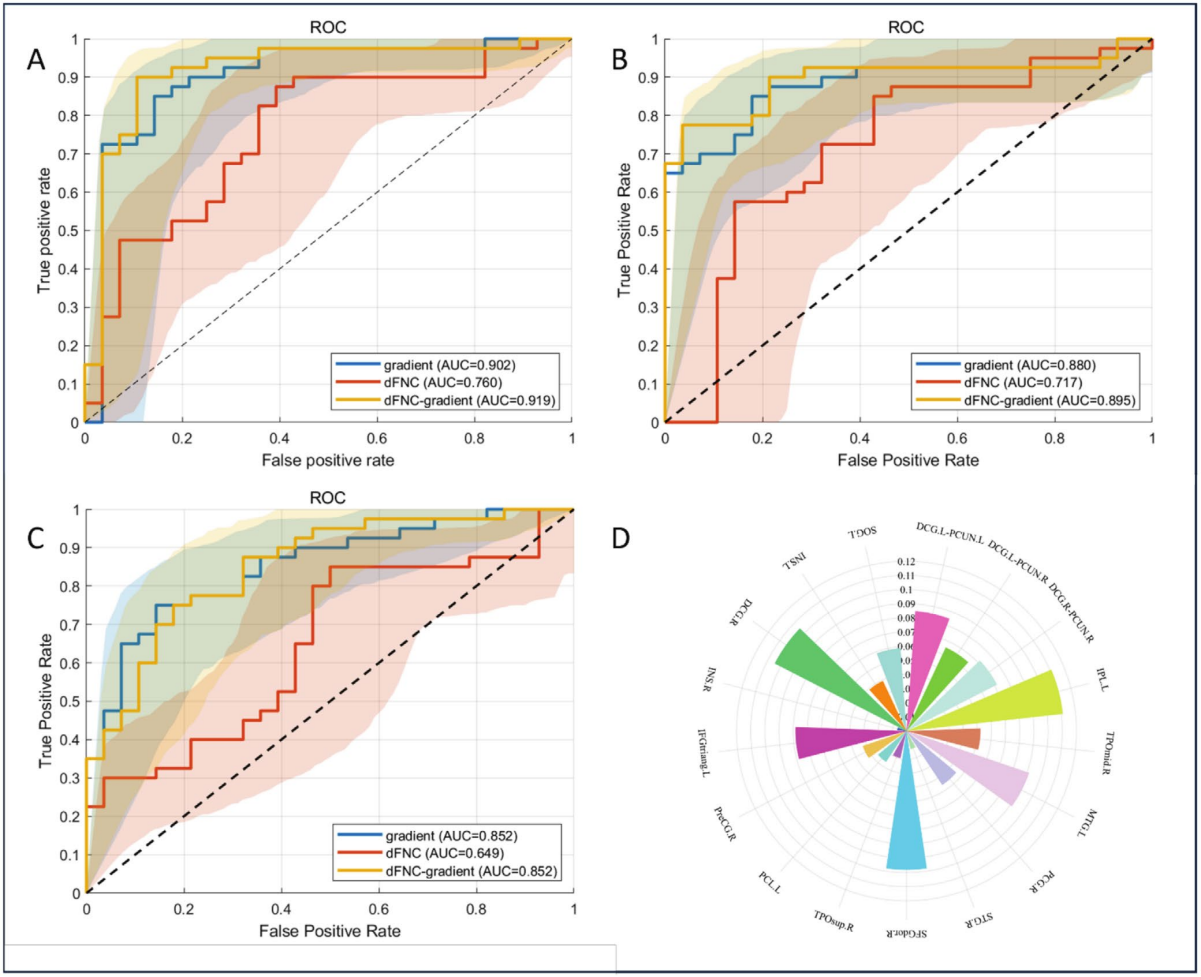
Predictive discrimination performance of various machine learning models in males. (A) The AUC‐ROC curves of the LinearSVM model based on three feature sets in males. (B) The AUC‐ROC curves of the RBFSVM model based on three feature sets in males. (C) The AUC‐ROC curves of the RF model based on three feature sets in males. (D) Given the significant differences in functional gradients and dynamic functional connectivity between the male ASD and TD groups, the radar chart illustrates each brain region's discriminative power. The key feature was the first gradient of the DAN in the left inferior parietal lobule, but the supramarginal and angular gyri.

**FIGURE 10:**
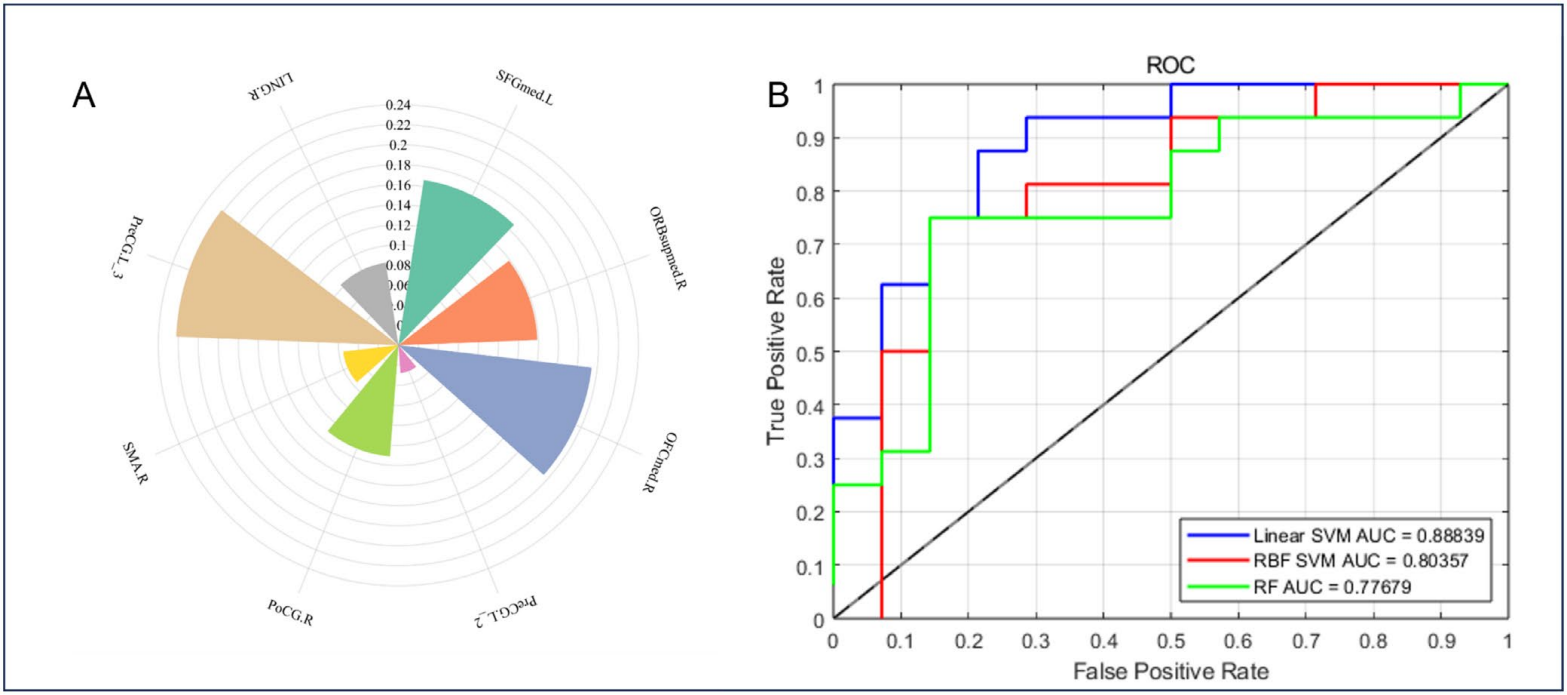
Predictive discrimination performance of various machine learning models (A) A Given the significant differences in functional gradients between the female ASD and TD groups, the radar chart illustrates each brain region's discriminative power. The key feature was the third gradient of the SMN in the left precentral gyrus. (B) The AUC‐ROC curves generated by the three machine learning models.

Given the sex‐specific differences in functional gradients and dynamic functional connectivity, we evaluated the predictive value of these features for distinguishing ASD from TD separately in males and females. The findings revealed the third gradient of the SMN—located in the left precentral gyrus—was the key feature for distinguishing female ASD from female TD (Figure 10A). Similarly, the first gradient of the DAN—located in the left inferior parietal lobule, but the supramarginal and angular gyri—was the key feature for distinguishing male ASD from male TD (Figure 9D). The AUC‐ROC curves for the three machine learning models are shown in Figures 9A–C and 10B, and the area under the curve, sensitivity, specificity, and accuracy are presented in Table 5.

**TABLE 5:** Performance metrics of various machine learning models.

			Linear_SVM	RBF_SVM	RF
Male	Gradient	AUC (%)	90.18	88.04	85.18
		Sensitivity (%)	80.00	85.00	85.00
		Specificity (%)	85.71	82.14	64.29
		Accuracy (%)	82.35	83.82	76.47
	dFNC	AUC (%)	75.98	71.70	64.91
		Sensitivity (%)	75.00	75.00	80.00
		Specificity (%)	64.29	57.14	53.57
		Accuracy (%)	70.59	67.65	69.12
	dFNC‐gradient	AUC (%)	91.88	89.46	85.18
		Sensitivity (%)	87.50	87.50	82.50
		Specificity (%)	89.29	78.57	67.86
		Accuracy (%)	88.24	83.82	76.47
Female	Gradient	AUC (%)	86.61	76.67	73.21
		Sensitivity (%)	87.50	93.75	71.43
		Specificity (%)	85.71	57.14	75.00
		Accuracy (%)	86.67	81.25	73.33

Abbreviations: AUC, area under the curve; Linear_SVM, linear support vector machine; RBF_SVM, radial basis function support vector machine; RF, random fores.

#### 3.3.4 Correlations Between Functional Gradients and Clinical Variables in Males

**FIGURE 11:**
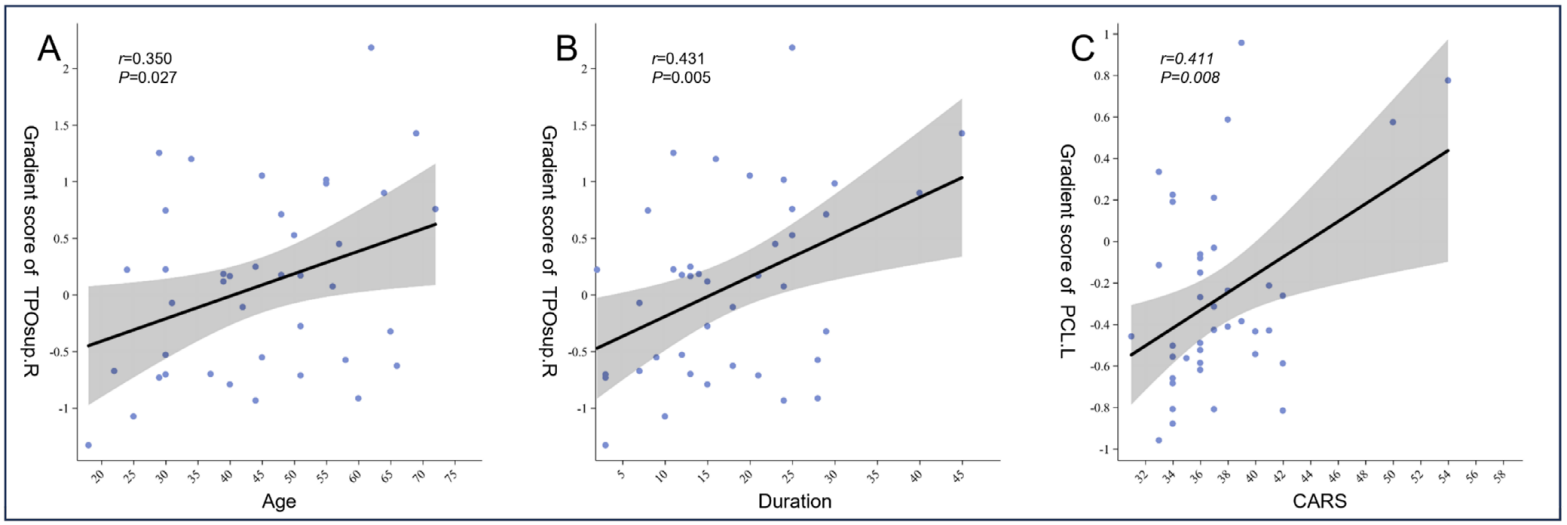
(A) Pearson correlation between the gradient score of the right temporal pole (superior temporal gyrus) and age in ASD patients; (B) Pearson correlation between the gradient score of the right temporal pole (superior temporal gyrus) and duration in ASD patients; (C) Pearson correlation between the gradient score of the left paracentral gyrus and CARS in ASD patients.

No significant correlations were found between the functionally abnormal brain regions in female participants and clinical variables. In the LIM, the right temporal pole (superior temporal gyrus) exhibited significant intergroup differences in functional gradient scores between the ASD and TD groups. These scores were positively correlated with age (*r* = 0.350, *p* = 0.027) (Figure 11A) and disease duration (*r* = 0.431, *p* = 0.005) (Figure 11B) in the ASD group. Additionally, in the SMN, the scores of the left paracentral lobule showed a positive correlation with CARS scores (*r* = 0.411, *p* = 0.008) (Figure 11C).

## 4 Discussion

This study systematically revealed significant abnormal patterns and sex differences in functional gradients and dFC in preschool male and female children with ASD. Notably, the study found that the right median cingulate and paracingulate gyri exhibited significant abnormalities in both functional gradients and dynamic functional connectivity, suggesting that they may serve as core pathological nodes of ASD. This finding indicates that the right median cingulate gyrus may act as a key region for cross‐network functional integration and dynamic regulation in ASD, with its abnormalities potentially producing cascading effects across multiple functional domains, including sensory input, attentional control, and social cognition. Male and female ASD children showed clear differences in the level of functional gradient abnormalities, with abnormalities in male ASD children primarily concentrated in the first and second gradients, while those in female ASD children were mainly concentrated in the third gradient. This suggests that different information processing pathways and neural mechanisms may be involved in ASD across sexes.

Firstly, in the functional gradients, the study revealed significant abnormalities in sensory‐motor, attentional, and social information processing in ASD children. Our results showed that the gradient value of the left paracentral lobule (primary motor cortex) was reduced, which may impair the precision of sensory‐motor integration, leading to stereotyped behaviors (e.g., repetitive hand clapping) and motor coordination deficits [35]. In addition, the reduced functional gradient in the left paracentral lobule was positively correlated with CARS scores, suggesting that functional abnormalities in the sensory‐motor pathway may be directly linked to core symptoms of ASD, such as stereotyped behaviors and motor coordination deficits. Higher CARS scores may reflect more severe stereotyped behaviors and poorer motor coordination, indicating that future interventions may need to target improving sensory‐motor integration to alleviate core ASD symptoms. Moreover, the increased gradient score in the right temporal pole (superior temporal gyrus) may reflect excessive clustering of local neural activity or be associated with sensory hypersensitivity (e.g., auditory or visual hypersensitivity) and abnormal processing of social information (e.g., heightened sensitivity to prosody) in ASD [36]. The positive correlation between the gradient score and both age and disease duration suggests that this abnormality may worsen with neurodevelopmental progression or represent a compensatory remodeling mechanism (e.g., enhancing local processing to compensate for deficits in global integration).

In the analysis of functional gradients, this study identified significant sex differences in the levels of abnormalities between male and female ASD children. Functional gradient abnormalities in male ASD children were primarily concentrated in the first and second gradients, suggesting impairments in sensory‐motor and cross‐network integration functions. The first and second gradients represent the hierarchical organization from low‐level sensory and motor functions to high‐level task and cognitive functions [37, 38]. Decreased functional gradient values in the first and second gradients of the DAN and SMN suggest structural damage in sensory‐motor integration and attentional regulation in male ASD patients. In addition, enhanced functional gradient values in the first gradient of the (LIM) may indicate excessive local processing and insufficient global integration during social cognition, which could lead to perceptual bias and communication difficulties in social interactions [39]. This pattern reflects the “Extreme Male Brain Theory,” which predicts “perception‐systematization” dysfunction, highlighting specific vulnerabilities in executive function and sensory‐motor control in male ASD patients [19].

In contrast, functional gradient abnormalities in female ASD children were mainly concentrated in the third gradient, suggesting impairments in local network characteristics and internal information integration. The third gradient is typically involved in self‐referential thinking, social interaction, and emotional regulation [40]. Abnormalities in the third gradient of the SMN, LIM, and VIS in female ASD patients may reflect deficits in social understanding and self‐awareness. This localized abnormal pattern suggests that female ASD patients may compensate for network dysfunction by enhancing emotional regulation and social communication strategies, consistent with the “Female Protective Effect” [41].

In terms of the classification ability of brain regions, the study found that the best discriminative region for male ASD patients was the left inferior parietal lobule in the DAN. The left inferior parietal lobule plays an important role in cross‐network information integration and attentional control [42]. A decrease in its functional gradient may cause ASD children to exhibit “excessive local attention” and “insufficient global information extraction” during information processing, leading to deficits in attentional allocation and task‐switching ability [43]. This finding is consistent with the typical deficits in executive function and attentional regulation observed in male ASD patients [44]. The best discriminative region for female ASD patients was the left precentral gyrus in the SMN. The left precentral gyrus plays a central role in motor planning and sensory feedback [45]. Enhanced functional gradient values in this region suggest that female ASD children may exhibit “excessive local reinforcement” in motor coordination and sensory feedback, leading to behavioral rigidity and heightened sensory sensitivity. This finding is highly consistent with the typical presentation of stereotyped behaviors and sensory abnormalities in female ASD children [46].

Dynamic functional connectivity analysis showed that hyperconnectivity between the right median cingulate gyrus and the precuneus in state 1 was significantly increased, suggesting that network activity in ASD children exhibits abnormal rigidity at rest. This dynamic rigidity may reflect deficits in executive function and cognitive flexibility in ASD children due to the inability to effectively adjust for prediction errors during task switching or environmental changes [47]. Enhanced coupling between the cingulate gyrus and the precuneus (DMN node) may represent a compensatory mechanism, where increased coupling with the DMN compensates for regulatory failure in the VAN–DMN dynamic balance [48]. However, over‐reliance on DMN‐dominated state 1 may hinder flexible switching to other states (e.g., state 2 or state 3), directly contributing to deficits in cognitive flexibility in ASD patients. According to the Predictive Coding Theory [49], ASD patients tend to rely on fixed internal models (corresponding to the stability of state 1) and have difficulty updating predictions based on new information (corresponding to the need for state switching). This mechanism may explain the tendency of ASD children to resist new environments and engage in repetitive behaviors. Functional gradient abnormalities in the right median cingulate and paracingulate gyri may reinforce this dynamic rigidity, suggesting a synergistic effect between functional gradient abnormalities and dynamic functional connectivity rigidity in ASD children.

In contrast to male participants, no significant correlations were observed between functional gradient or dFC measures and clinical scores in females with ASD. This lack of association may reflect sex‐specific protective or compensatory mechanisms. Prior research has proposed a female protective effect, suggesting that females may require a higher neurobiological burden for ASD expression [50]. In addition, studies have reported enhanced fronto‐amygdala and limbic–prefrontal connectivity in females with ASD, which may serve as compensatory mechanisms to buffer symptom severity [15], particularly during early development [51]. These mechanisms may obscure direct associations between brain function and symptom severity, especially in small samples. Given the limited number of female ASD participants (*n* = 14), all sex‐specific findings in this study should be considered exploratory, and future research with larger, sex‐balanced cohorts is needed to determine whether these observations reflect true neurobiological differences or sampling variability.

### 4.1 Strengths and Limitations

Utilizing functional gradient and dynamic functional connectivity analyses, this study identified concurrent abnormalities in spatial hierarchical integration and temporal dynamic coordination in the brains of preschool‐aged children with autism spectrum disorder (ASD). Furthermore, pronounced sex‐related differences were observed in these functional features between boys and girls with ASD, offering a potential neuroimaging mechanism that may underlie the markedly higher prevalence of ASD in males. We further propose that the left inferior parietal lobule may serve as potential early neuroimaging biomarkers in males. By breaking away from traditional single‐gradient analysis, this approach may uncover separate impairments in information processing mechanisms across different hierarchical levels, offering a new explanatory framework for the heterogeneity of ASD.

However, there are limitations in this study. First, existing research primarily focuses on brain developmental atlases for infants aged 0–2 years and children aged 6–12 years, while brain templates for children aged 2–6 years have not been widely established. Therefore, this study employed an adult MNI template for spatial normalization. While this practice is commonly used in pediatric fMRI research, applying adult templates to young children can introduce registration bias—particularly in rapidly developing cortical regions such as the prefrontal cortex and temporal pole. Such bias may reduce voxel‐level alignment accuracy and, in turn, affect the estimation of functional gradients and dFC metrics. To address this, we applied advanced registration techniques and conducted thorough visual inspections of alignment quality. Nonetheless, we acknowledge this limitation. In future work, we plan to recruit a larger cohort of typically developing children aged 2–6 years to construct age‐appropriate pediatric brain templates and prioritize the inclusion of participants who do not require sedation, thereby minimizing potential sources of bias. Future studies will aim to recruit a larger cohort of typically developing children aged 2–6 years to establish usable child brain templates and include patients who do not require sedative medications to minimize bias. The use of sedative medications during MRI scanning in a subset of participants may have introduced potential confounds in this study. Sedatives administered during image acquisition can alter neural activity and functional connectivity, potentially impacting the estimation of functional gradients. In a retrospective analysis of chloral hydrate use across various pediatric neurofunctional assessments, Fong et al. reported higher sedation failure rates under certain conditions and suggested that chloral hydrate may interfere with neurofunctional measures such as EEG and MRI [52]. Similarly, Qi et al. found that mild sedation with 30 mg/kg of chloral hydrate reduced the number of activated voxels in the visual cortex and attenuated BOLD signal amplitudes, although these effects did not reach statistical significance [53]. Thus, some of the observed alterations in brain network functional gradients in this study may be partially attributable to the influence of sedative agents. Future studies will aim to increase the sample size and preferentially include participants who do not require sedation, thereby minimizing its potential confounding effects on neuroimaging findings. Additionally, as this is a single‐center study, larger multi‐center studies are needed to further validate the reliability and stability of these findings.

## 5 Conclusion

Preschool‐aged boys and girls with ASD exhibit significant sex differences in functional gradients and dynamic functional connectivity, underscoring the complexity and heterogeneity of ASD. These findings provide a novel theoretical framework for understanding the neuroimaging mechanisms underlying ASD.

## Conflicts of Interest

The authors declare no conflicts of interest.

## Supporting information


**Data S1:** cns70562‐sup‐0001‐Supinfo.docx.

## Data Availability

The DIBS MRI datasets generated and analyzed during the current study are not publicly available because of data privacy regulations of patient data; but are available from the corresponding author upon reasonable request after approval of the local data protection office.
