# Supplementary material for: Sex Differences in Functional Gradients and Dynamic Functional Connectivity in Preschool‐Aged Children With ASD
Source: CNS Neurosci Ther. 2025 Aug 10;31(8):e70562. doi: 10.1111/cns.70562 (PMC12336372; doi:10.1111/cns.70562)
Supplement: Supplementary file 1 — Data S1: cns70562‐sup‐0001‐Supinfo.docx. [file CNS-31-e70562-s001.docx]

key intermediate files (feature lists, confusion matrices, train/test splits)

feature lists:[gradient_dFC]

DAN_gradient1_Parietal_Inf_L

DAN_gradient1_Temporal_Pole_Mid_R

DMN_gradient1_Temporal_Mid_L

DMN_gradient1_Cingulate_Post_R

DMN_gradient1_Temporal_Sup_R

DMN_gradient2_Frontal_Sup_2_R

LIM_gradient1_Temporal_Pole_Sup_R

SMN_gradient2_Paracentral_Lobule_L

SMN_gradient2_ Precentral_R

VAN_gradient1_ Frontal_Inf_Tri_L

VAN_gradient1_ Insula_R

VAN_gradient1_ Cingulate_Mid_R

VAN_gradient2_Insula_L

VIS_gradient3_Occipital_Sup_L

State1_dfc_left median cingulate and paracingulate gyri with left precuneus gyrus

State1_dfc_left median cingulate and paracingulate gyri with right precuneus gyrus

State1_dfc_right median cingulate and paracingulate gyri with right precuneus gyrus

Table S1 Classification performance of LinearSVM model based on three feature sets in males

| FeatureSet | AUC_Mean | AUC_CI_Low | AUC_CI_High | Accuracy | Sensitivity | Specificity | AUC_Curve |
| --- | --- | --- | --- | --- | --- | --- | --- |
| gradient | 0.99810049 | 0.979166667 | 1 | 82.35% | 80.00% | 85.71% | 90.18% |
| dFNC | 0.930646008 | 0.84 | 1 | 70.59% | 75.00% | 64.29% | 75.98% |
| dFNC_gradient | 0.999632353 | 1 | 1 | 88.24% | 87.50% | 89.29% | 91.88% |


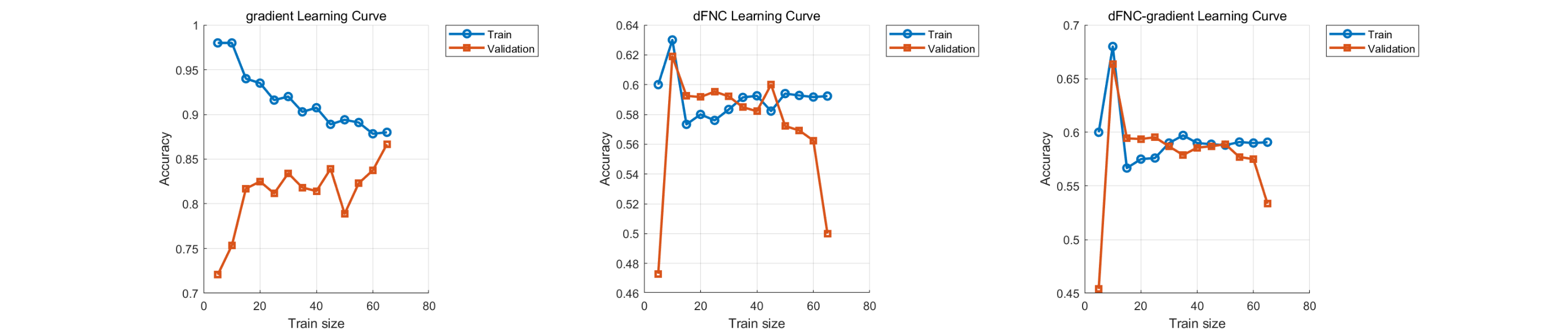


Fig 1 Learning curves of the LinearSVM model for binary classification of male autism spectrum disorder based on three different feature types. The x-axis represents the training set size, and the y-axis represents classification accuracy.


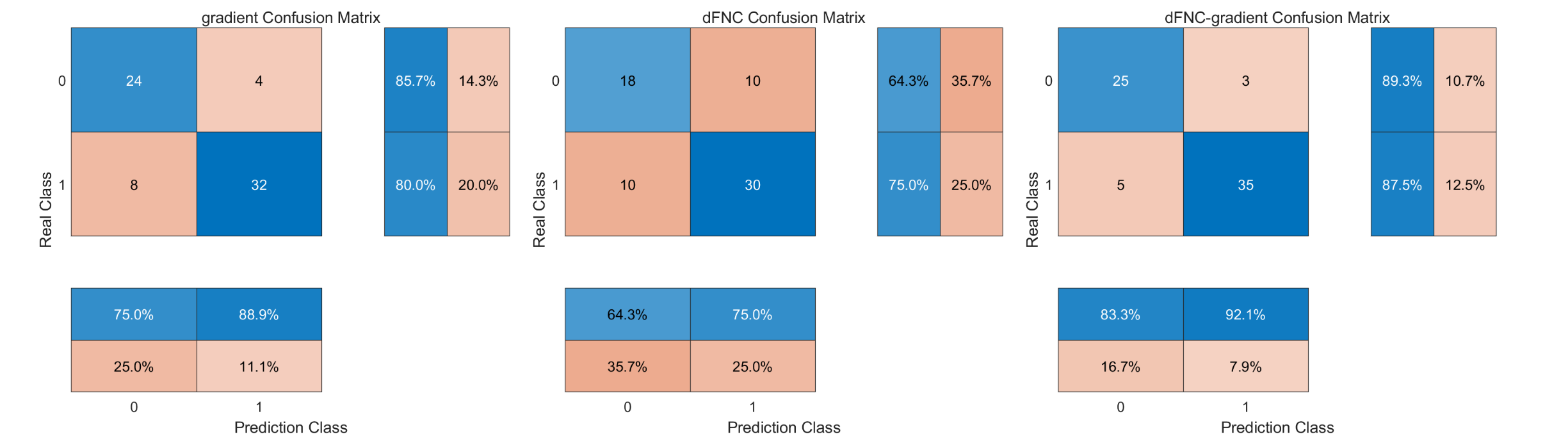
fig 2 Confusion matrices of the LinearSVM model using three different feature types for binary classification in male autism spectrum disorder. Each confusion matrix shows the correspondence between true and predicted classes, presented in both absolute numbers and percentages.

Table S2 Classification performance of RBFSVM model based on three feature sets in males

| FeatureSet | AUC_Mean | AUC_CI_Low | AUC_CI_High | Accuracy | Sensitivity | Specificity | AUC_Curve |
| --- | --- | --- | --- | --- | --- | --- | --- |
| gradient | 0.997058824 | 0.975 | 1 | 83.82% | 85.00% | 82.14% | 88.04% |
| dFNC | 0.93040091 | 0.84 | 1 | 67.65% | 75.00% | 57.14% | 71.70% |
| dFNC_gradient | 0.999019608 | 0.979166667 | 1 | 83.82% | 87.50% | 78.57% | 89.46% |


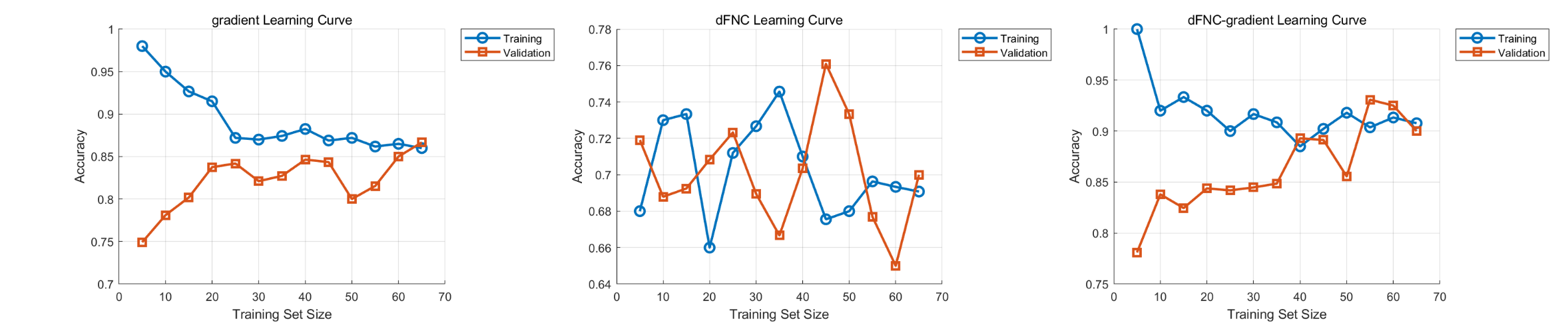


Fig 3 Learning curves of the RBFSVM model for binary classification of male autism spectrum disorder based on three different feature types. The x-axis represents the training set size, and the y-axis represents classification accuracy.


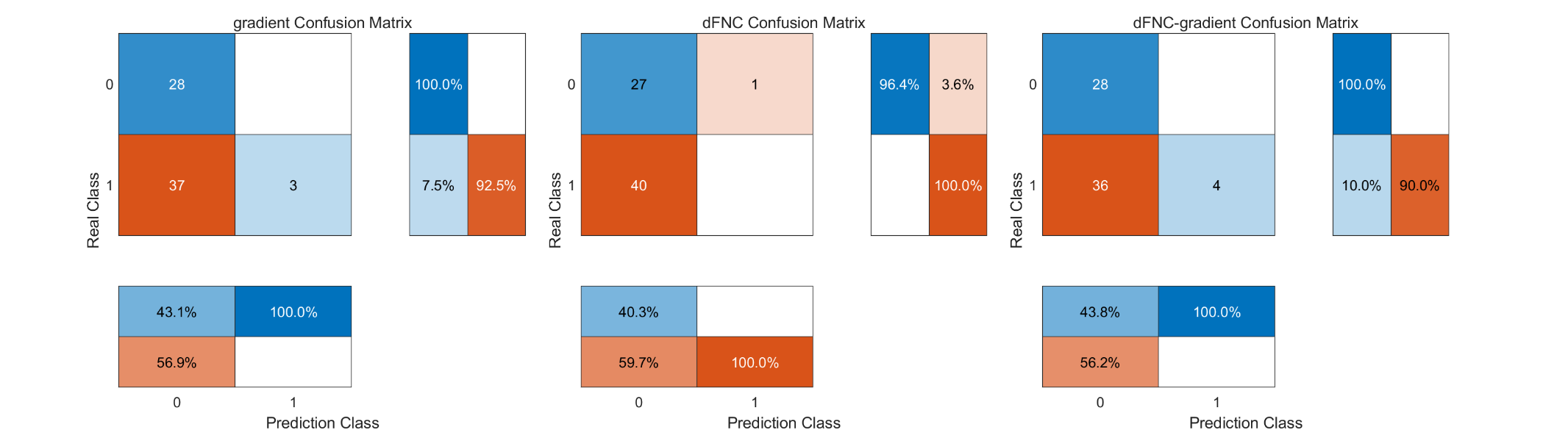


fig 4 Confusion matrices of the RBFSVM model using three different feature types for binary classification in male autism spectrum disorder. Each confusion matrix shows the correspondence between true and predicted classes, presented in both absolute numbers and percentages.

Table S3 Classification performance of RF model based on three feature sets in males

| FeatureSet | AUC_Mean | AUC_CI_Low | AUC_CI_High | Accuracy | Sensitivity | Specificity | AUC_Curve |
| --- | --- | --- | --- | --- | --- | --- | --- |
| gradient | 0.996577381 | 0.956904762 | 1 | 76.47% | 85.00% | 64.29% | 85.18% |
| dFNC | 0.918679972 | 0.8125 | 1 | 69.12% | 80.00% | 53.57% | 64.91% |
| dFNC_gradient | 0.999693627 | 1 | 1 | 76.47% | 82.50% | 67.86% | 85.18% |


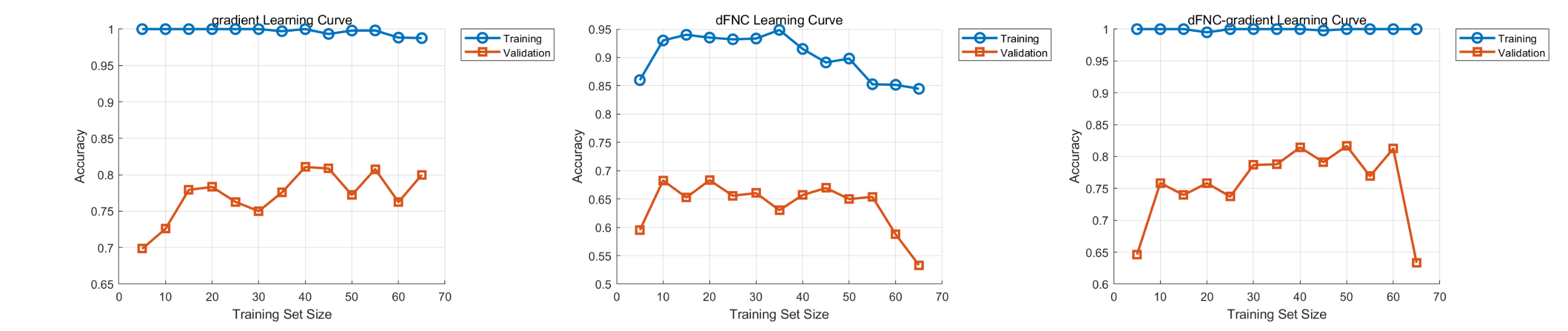


Fig 5 Learning curves of the RF model for binary classification of male autism spectrum disorder based on three different feature types. The x-axis represents the training set size, and the y-axis represents classification accuracy.

.


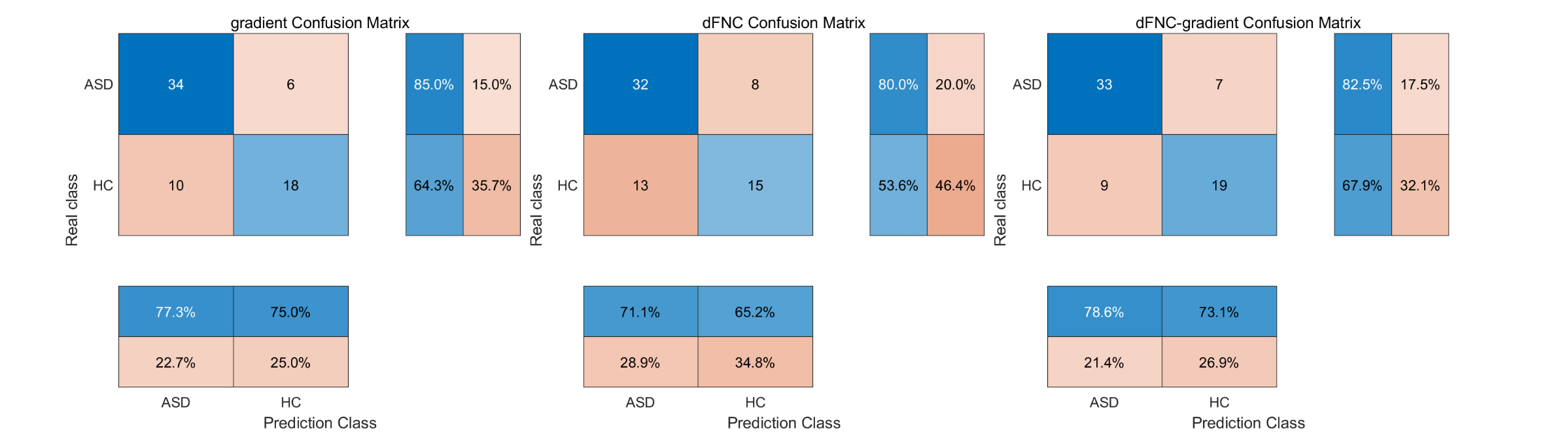


Fig 6 Confusion matrices of the RF model using three different feature types for binary classification in male autism spectrum disorder. Each confusion matrix shows the correspondence between true and predicted classes, presented in both absolute numbers and percentages.

Table S4 Classification performance of LinearSVM model based on gradient features in females

| FeatureSet | AUC_Mean | AUC_CI_Low | AUC_CI_High | Accuracy | Sensitivity | Specificity | AUC_Curve |
| --- | --- | --- | --- | --- | --- | --- | --- |
| gradient | 1 | 1 | 1 | 86.67% | 87.50% | 85.71% | 86.61% |


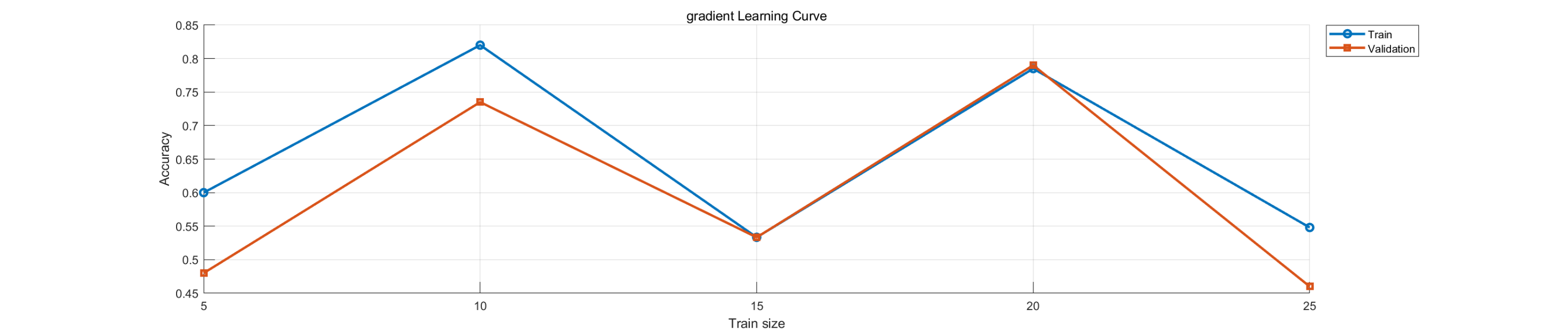


Fig 7 Learning curves of the LinearSVM model for binary classification of female autism spectrum disorder based on one feature types. The x-axis represents the training set size, and the y-axis represents classification accuracy.


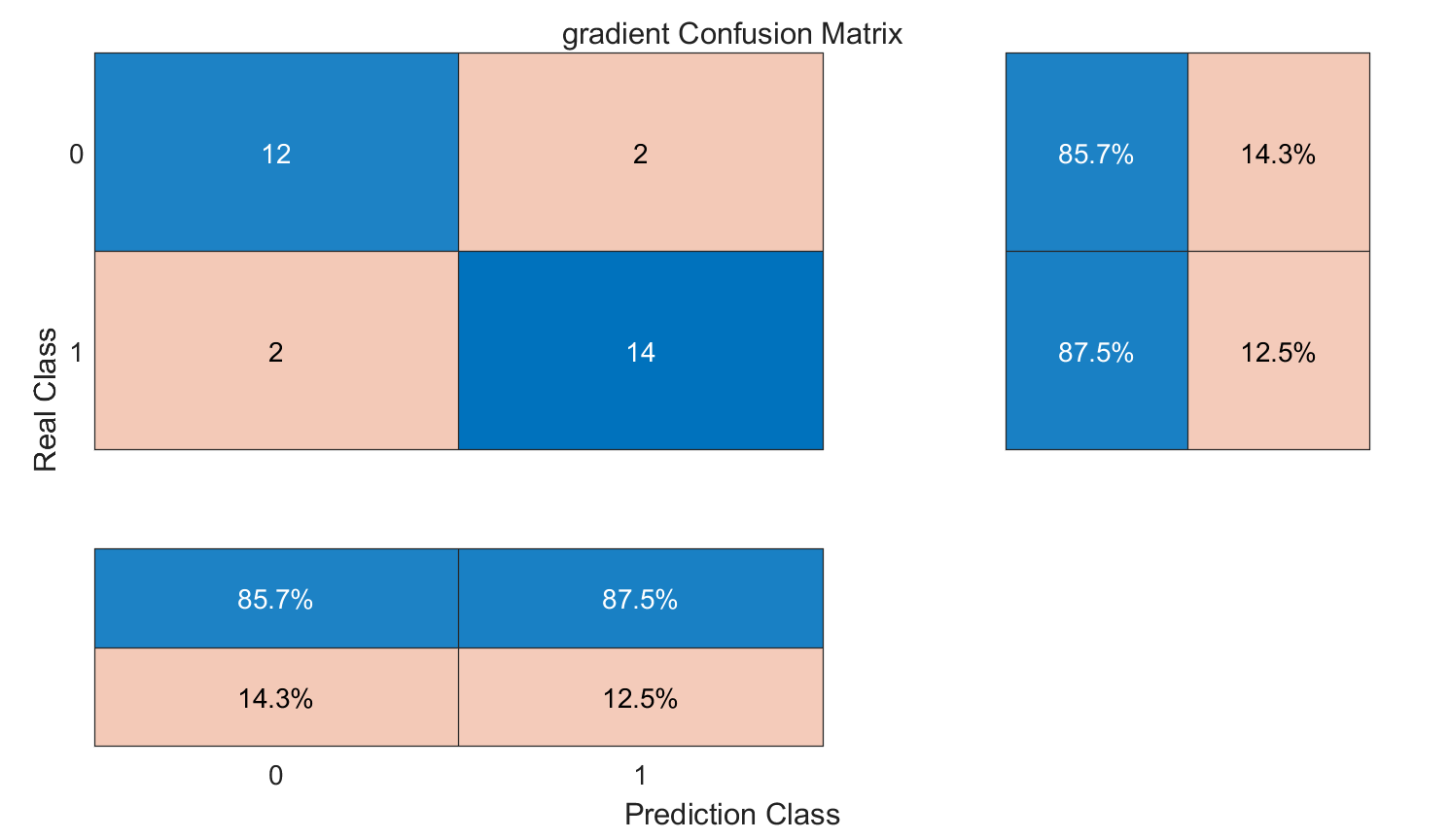


Fig 8 Confusion matrices of the LinearSVM model using one feature types for binary classification in female autism spectrum disorder. Each confusion matrix shows the correspondence between true and predicted classes, presented in both absolute numbers and percentages.

Table S5 Classification performance of RBFSVM model based on gradient features in females

| FeatureSet | AUC_Mean | AUC_CI_Low | AUC_CI_High | Accuracy | Sensitivity | Specificity | AUC_Curve |
| --- | --- | --- | --- | --- | --- | --- | --- |
| gradient | 1 | 1 | 1 | 76.67% | 93.75% | 57.14% | 81.25% |


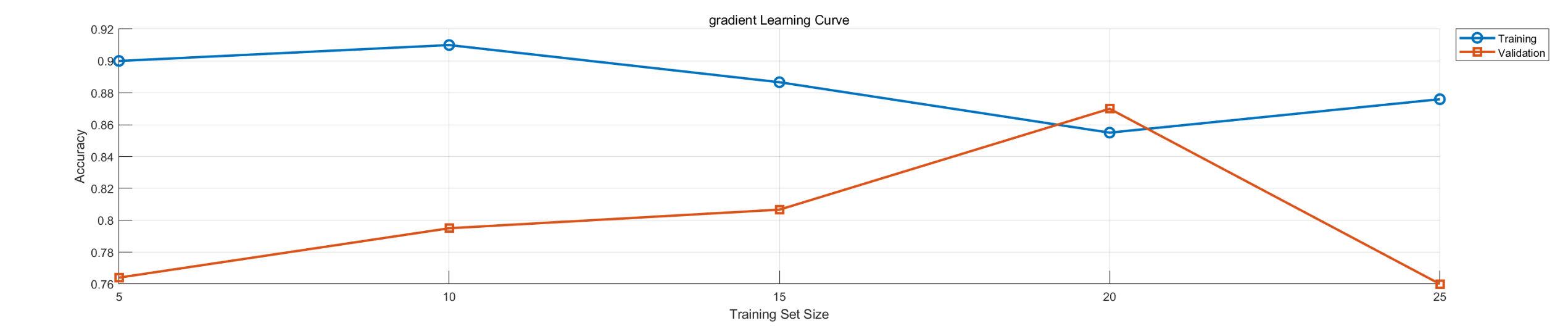


fig 9 Learning curves of the RBFSVM model for binary classification of female autism spectrum disorder based on one feature types. The x-axis represents the training set size, and the y-axis represents classification accuracy.


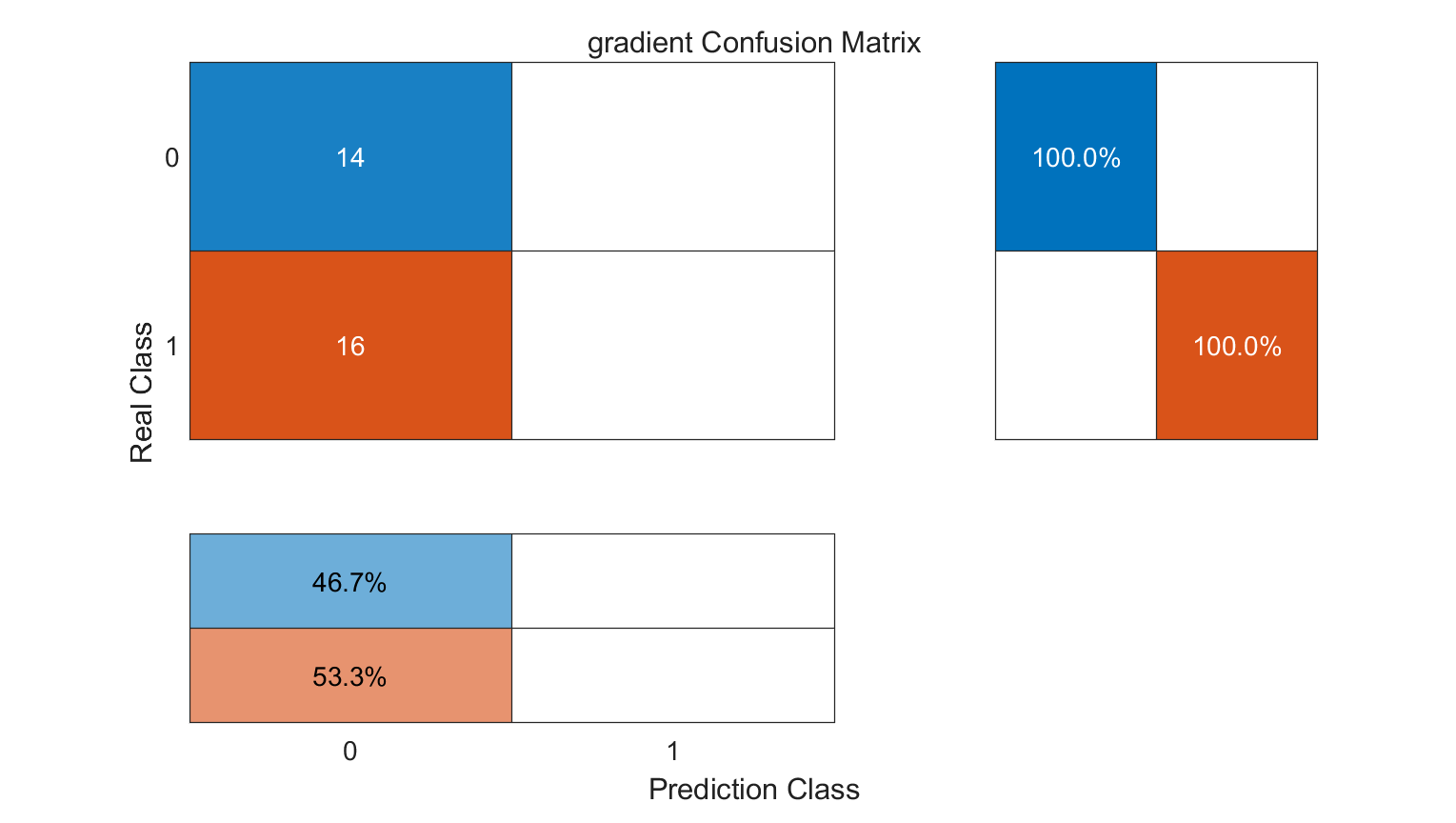


Fig 10 Confusion matrices of the RBFSVM model using one feature types for binary classification in female autism spectrum disorder. Each confusion matrix shows the correspondence between true and predicted classes, presented in both absolute numbers and percentages.

Table S6 Classification performance of RF model based on gradient features in females

| FeatureSet | AUC_Mean | AUC_CI_Low | AUC_CI_High | Accuracy | Sensitivity | Specificity | AUC_Curve |
| --- | --- | --- | --- | --- | --- | --- | --- |
| gradient | 1 | 1 | 1 | 73.33% | 75.00% | 71.43% | 73.21% |


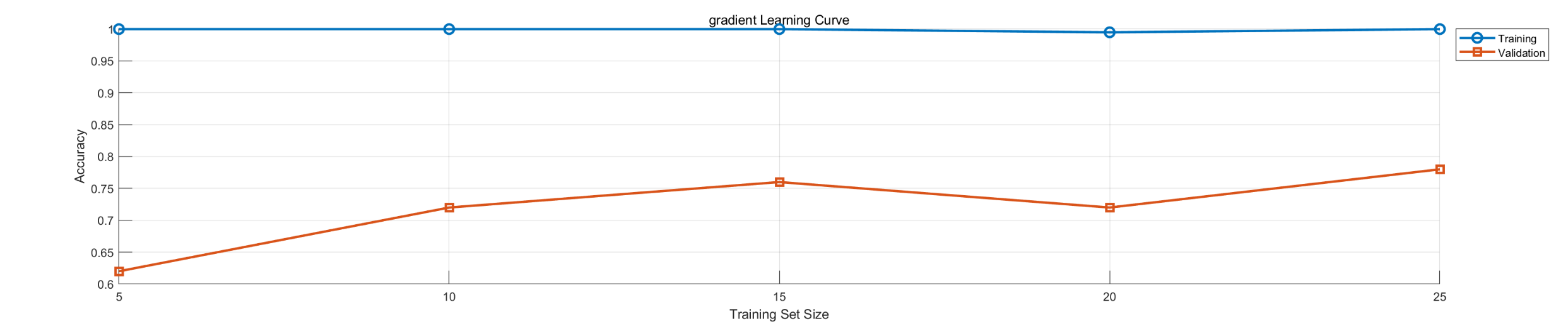


fig 11 Learning curves of the RF model for binary classification of female autism spectrum disorder based on one feature types. The x-axis represents the training set size, and the y-axis represents classification accuracy.


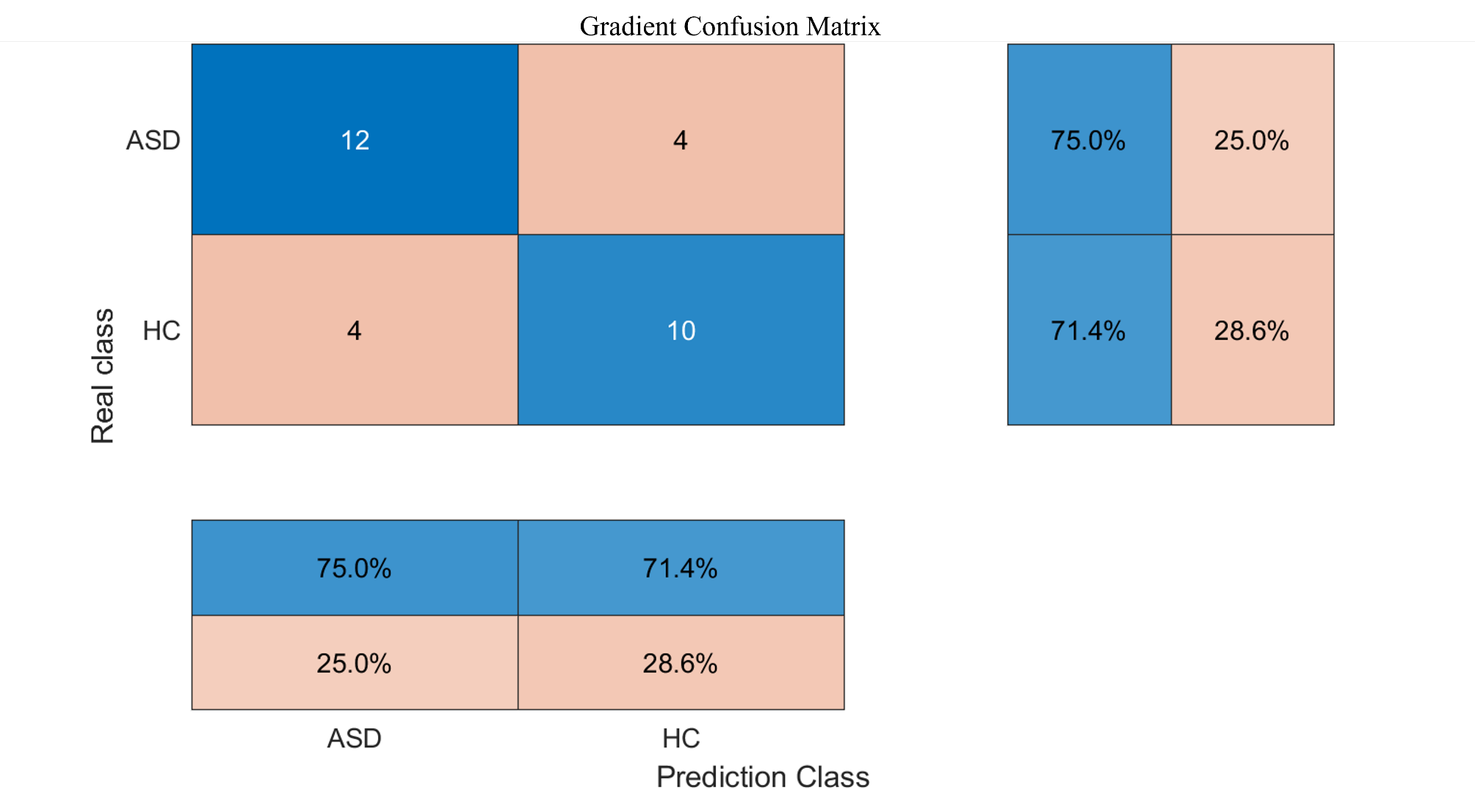


Fig 12 Confusion matrices of the RF model using one feature types for binary classification in female autism spectrum disorder. Each confusion matrix shows the correspondence between true and predicted classes, presented in both absolute numbers and percentages.

Table S7 Lists of all significant clusters

|  |  |  | Brain regions  (AAL) | Voxels, n | MNI coordinates, mm  （x, y, z） | | | | Peak t values | *P* values |
| --- | --- | --- | --- | --- | --- | --- | --- | --- | --- | --- |
| Male | DAN |  |  |  |  | |  |  |  |  |
|  |  | Gradient 1 | Parietal_Inf_L | 306 | -27 | -75 | | 45 | -4.1378 | 0.000282 |
|  |  |  | Temporal_Pole_Mid_R | 223 | 54 | 9 | | -36 | 3.3581 | 0.002246 |
|  | LIM |  |  |  |  |  | |  |  |  |
|  |  | Gradient 1 | Temporal_Pole_Sup_R | 225 | 54 | 15 | | -15 | 3.4848 | 0.001603 |
|  | SMN |  |  |  |  |  | |  |  |  |
|  |  | Gradient 2 | Paracentral_Lobule_L | 1062 | -12 | -39 | | 78 | -4.7304 | 0.000058 |
|  |  |  | Precentral_R | 615 | 48 | -12 | | 57 | 4.1554 | 0.000269 |
|  | VIS |  |  |  |  |  | |  |  |  |
|  |  | Gradient 3 | Occipital_Sup_L | 232 | -12 | -96 | | 9 | -3.4077 | 0.001968 |
|  | VAN |  |  |  |  |  | |  |  |  |
|  |  | Gradient 1 | Frontal_Inf_Tri_L | 386 | -30 | 30 | | 0 | 4.0334 | 0.000372 |
|  |  |  | Insula_R | 329 | 42 | 12 | | -3 | 3.4225 | 0.001892 |
|  |  |  | Cingulate_Mid_R | 212 | 9 | -33 | | 33 | -4.8423 | 0.000043 |
|  |  | Gradient 2 | Insula_L | 192 | -27 | 27 | | 6 | -2.8382 | 0.008966 |
|  | DMN |  |  |  |  |  | |  |  |  |
|  |  | Gradient 1 | Temporal_Mid_L | 682 | -48 | -51 | | 0 | -4.2004 | 0.000238 |
|  |  |  | Cingulum_Post_R | 537 | 3 | -54 | | 30 | 3.9370 | 0.000381 |
|  |  |  | Temporal_Sup_R | 445 | 51 | 0 | | -15 | -3.4780 | 0.001631 |
|  |  | Gradient 2 | Frontal_Sup_R | 397 | 15 | 51 | | 45 | 4.0357 | 0.000370 |
| Female | DMN |  |  |  |  |  | |  |  |  |
|  |  | Gradient 1 | Frontal_Sup_Medial_L | 194 | -3 | 48 | | 54 | -4.8404 | 0.000043 |
|  |  | Gradient 2 | Frontal_Med_Orb_R | 148 | 12 | 39 | | -12 | 3.9815 | 0.000427 |
|  | SMN |  |  |  |  |  | |  |  |  |
|  |  | Gradient 2 | Precentral_L | 235 | -39 | -3 | | 36 | 3.7027 | 0.000897 |
|  |  | Gradient3 | Postcentral_R | 572 | 30 | -36 | | 48 | 3.9441 | 0.000472 |
|  |  |  | Supp_Motor_Area_R | 409 | 6 | 12 | | 54 | -4.3736 | 0.000150 |
|  |  |  | Precentral_L | 219 | -45 | -6 | | 39 | -3.9239 | 0.000498 |
|  | LIM |  |  |  |  |  | |  |  |  |
|  |  | Gradient 3 | OFCmed_R | 132 | 15 | 21 | | -24 | -5.0601 | 0.000024 |
|  | VIS |  |  |  |  |  | |  |  |  |
|  |  | Gradient 3 | Lingual_R | 308 | 18 | -78 | | 9 | 3.7039 | 0.000894 |


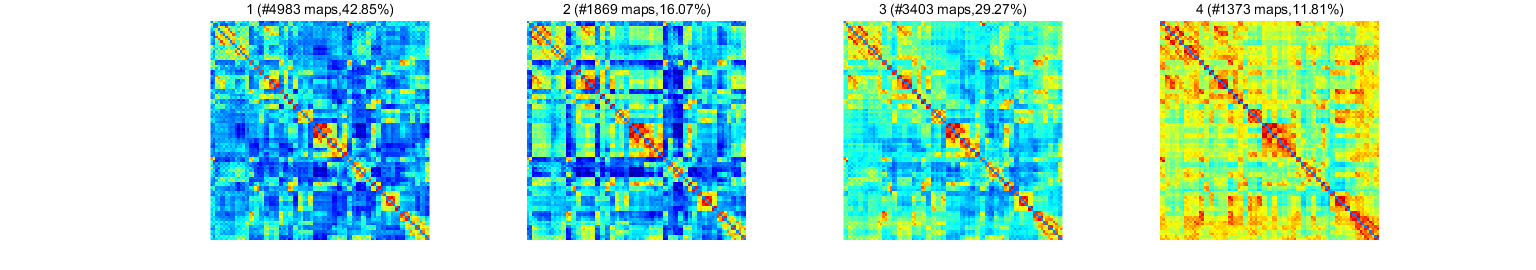
Compare results using windows of 30 TR, 80 TR, and step sizes of 2 TR, documenting whether state metrics and group differences remain consistent


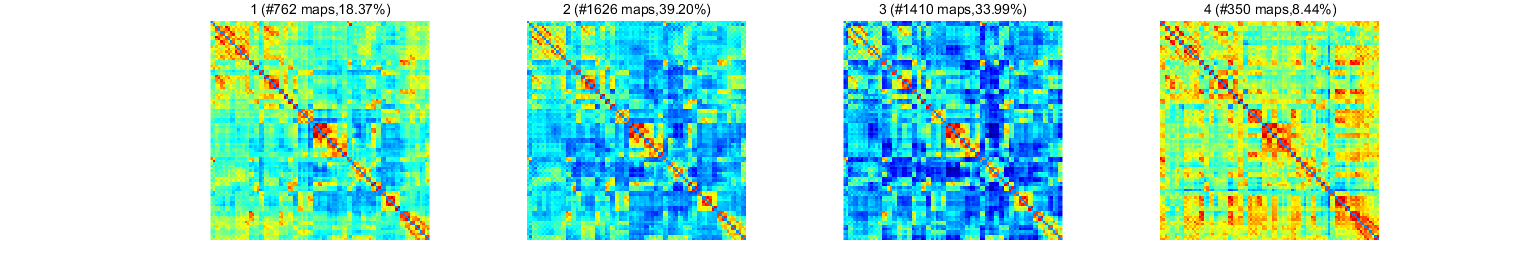
Fig 13 Brain feature clustering at k=4(windows of 30 TR and step sizes of 1 TR) in males

Fig 14 Brain feature clustering at k=4(windows of 80 TR and step sizes of 1 TR) in males


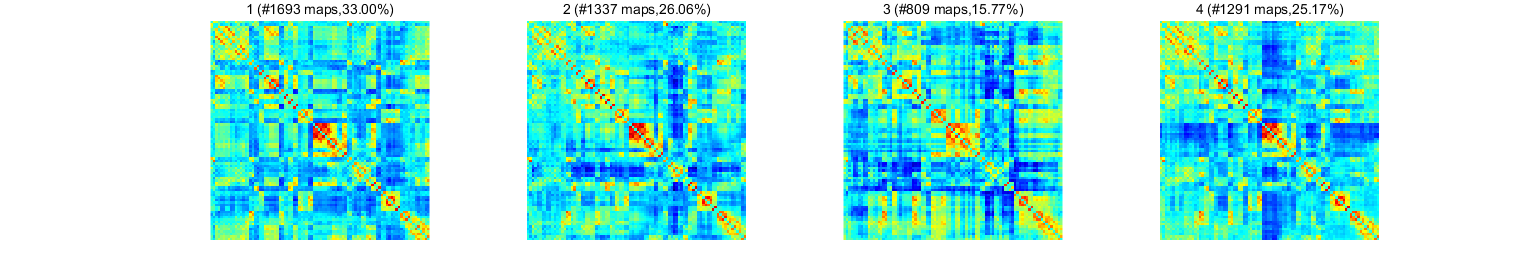
Fig 15 Brain feature clustering at k=4(windows of 50 TR and step sizes of 2 TR) in males
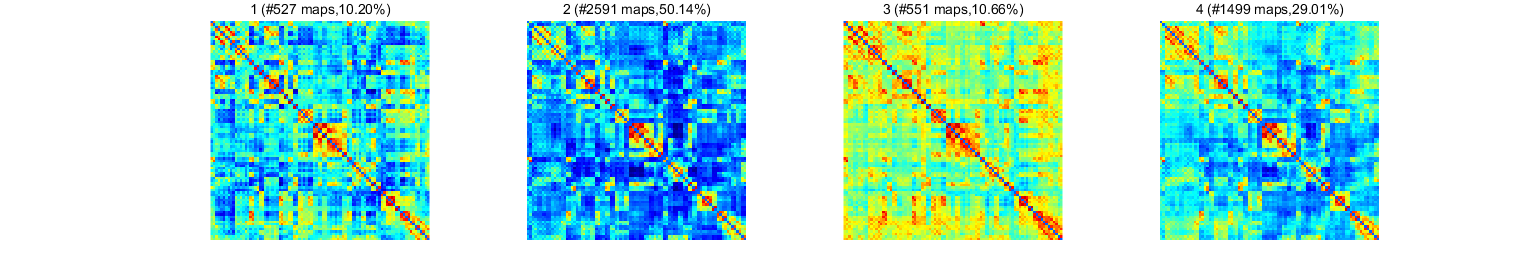


Fig 16 Brain feature clustering at k=4(windows of 30 TR and step sizes of 1 TR) in females


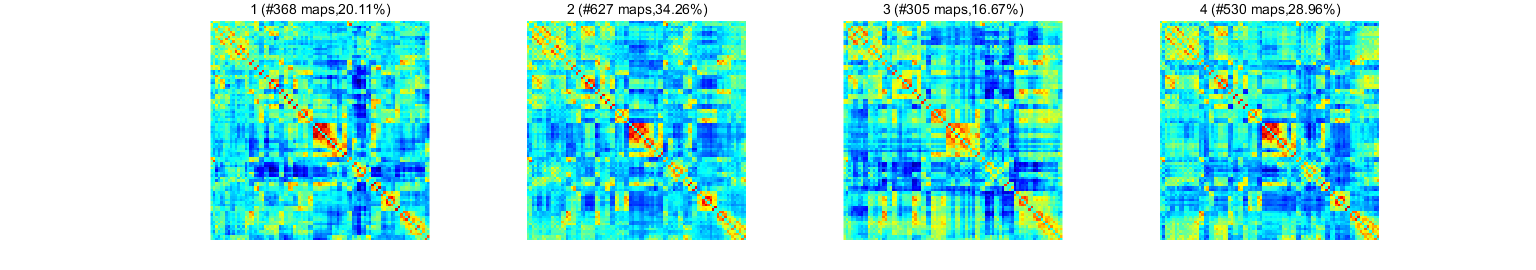
 Fig 17 Brain feature clustering at k=4(windows of 80 TR and step sizes of 1 TR) in females

Fig 18 Brain feature clustering at k=4(windows of 50 TR and step sizes of 2 TR) in females
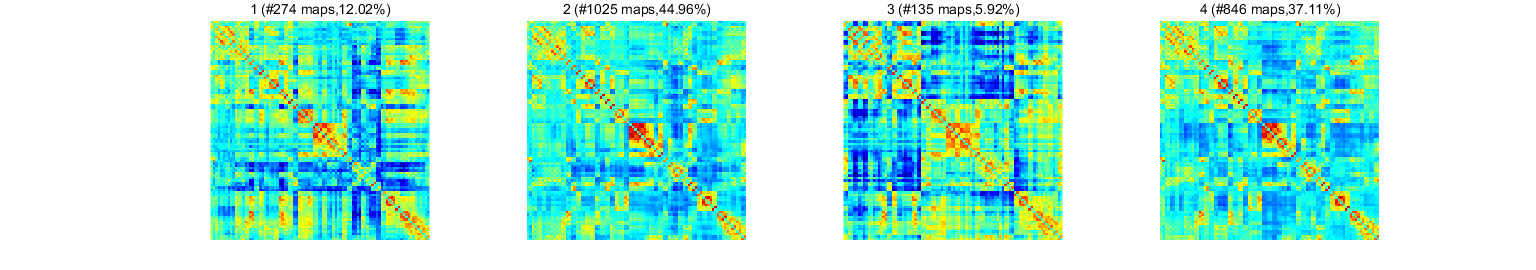


Result: We found that under these alternative settings, the group differences in dynamic functional connectivity between ASD patients and controls were no longer statistically significant. While 30–80 TR window lengths have been used in previous studies and are generally considered stable, we note that those studies typically involved larger sample sizes. Therefore, the parameter sensitivity observed in our study may reflect a dependence of sliding-window performance on sample size.

Female Gradient Calculations

Sensitivity analyses were performed using different connection thresholds (e.g., 5%, 15%, 20%) and alpha values (e.g., 0, 0.5, 1) to demonstrate the stability of the resulting gradients;

Threshold combinations 5%-0 5%-0.5 5%-1

15%-0 15%-0.5 15%-1

20%-0 20%-0.5 20%-1

Table S8 Functional gradients of seven brain networks in females under multiple parameter settings

| 参数 | 梯度 | 脑区 | MNI峰值坐标 | | | T value | P value | Cluster Size |
| --- | --- | --- | --- | --- | --- | --- | --- | --- |
|  |  |  | X | Y | Z |  |  |  |
| 01_VIS_percent80_A00 | gradient3 | Lingual_R | 21 | -87 | -3 | 4.542 | 0.000097 | 258 |
| 01_VIS_percent80_A05 | gradient3 | Lingual_R | 21 | -87 | -3 | 4.542 | 0.000097 | 258 |
| 01_VIS_percent80_A10 | gradient3 | Lingual_R | 21 | -87 | -3 | 4.542 | 0.000097 | 258 |
| 01_VIS_percent95_A00 | gradient2 | Lingual_L | -24 | -78 | -12 | -5.507 | 0.000007 | 911 |
|  |  | Occipital_Mid_R | 39 | -69 | 36 | 3.823 | 0.000674 | 895 |
| 01_VIS_percent95_A05 | gradient2 | Lingual_L | -24 | -78 | -12 | -5.507 | 0.000007 | 911 |
|  |  | Occipital_Mid_R | 39 | -69 | 36 | 3.823 | 0.000674 | 895 |
| 01_VIS_percent95_A10 | gradient2 | Lingual_L | -24 | -78 | -12 | -5.507 | 0.000007 | 911 |
|  |  | Occipital_Mid_R | 39 | -69 | 36 | 3.823 | 0.000674 | 895 |
| 02_SMN_percent85_A00 | gradient1 | Rolandic_Oper_R | 60 | 3 | 12 | 3.598 | 0.001221 | 662 |
|  |  | Postcentral_L | -54 | -15 | 42 | 3.700 | 0.000934 | 524 |
|  |  | Supp_Motor_Area_R | 3 | -9 | 75 | -4.341 | 0.000167 | 291 |
|  | gradient3 | Postcentral_R | 57 | -18 | 48 | -3.354 | 0.002299 | 372 |
|  |  | Supp_Motor_Area_L | -6 | -6 | 57 | 2.706 | 0.011464 | 209 |
|  |  | Postcentral_L | -51 | -18 | 57 | -3.746 | 0.000827 | 204 |
| 02_SMN_percent85_A05 | gradient1 | Rolandic_Oper_R | 60 | 3 | 12 | 3.598 | 0.001221 | 662 |
|  |  | Postcentral_L | -54 | -15 | 42 | 3.700 | 0.000934 | 524 |
|  |  | Supp_Motor_Area_R | 3 | -9 | 75 | -4.341 | 0.000167 | 291 |
|  | gradient3 | Postcentral_R | 57 | -18 | 48 | -3.354 | 0.002299 | 372 |
|  |  | Supp_Motor_Area_L | -6 | -6 | 57 | 2.706 | 0.011464 | 209 |
|  |  | Postcentral_L | -51 | -18 | 57 | -3.746 | 0.000827 | 204 |
| 02_SMN_percent85_A10 | gradient1 | Rolandic_Oper_R | 60 | 3 | 12 | 3.598 | 0.001221 | 662 |
|  |  | Postcentral_L | -54 | -15 | 42 | 3.700 | 0.000934 | 524 |
|  |  | Supp_Motor_Area_R | 3 | -9 | 75 | -4.341 | 0.000167 | 291 |
|  | gradient3 | Postcentral_R | 57 | -18 | 48 | -3.354 | 0.002299 | 372 |
|  |  | Supp_Motor_Area_L | -6 | -6 | 57 | 2.706 | 0.011464 | 209 |
|  |  | Postcentral_L | -51 | -18 | 57 | -3.746 | 0.000827 | 204 |
| 03_DAN_percent85_A00 | gradient3 | Temporal_Inf_R | 54 | 3 | -42 | 5.352 | 0.000011 | 263 |
|  |  | Parietal_Inf_L | -54 | -21 | 39 | 4.038 | 0.000379 | 173 |
| 03_DAN_percent85_A05 | gradient3 | Temporal_Inf_R | 54 | 3 | -42 | 5.352 | 0.000011 | 263 |
|  |  | Parietal_Inf_L | -54 | -21 | 39 | 4.038 | 0.000379 | 173 |
| 03_DAN_percent85_A10 | gradient3 | Temporal_Inf_R | 54 | 3 | -42 | 5.352 | 0.000011 | 263 |
|  |  | Parietal_Inf_L | -54 | -21 | 39 | 4.038 | 0.000379 | 173 |
| 03_DAN_percent95_A00 | gradient2 | Parietal_Inf_L | -54 | -30 | 48 | 4.335 | 0.000170 | 218 |
| 03_DAN_percent95_A05 | gradient2 | Parietal_Inf_L | -54 | -30 | 48 | 4.335 | 0.000170 | 218 |
| 03_DAN_percent95_A10 | gradient2 | Parietal_Inf_L | -54 | -30 | 48 | 4.335 | 0.000170 | 218 |
| 04_VAN_percent85_A00 | gradient3 | Cingulum_Mid_R | 15 | -12 | 45 | -4.236 | 0.000223 | 482 |
| 04_VAN_percent85_A05 | gradient3 | Cingulum_Mid_R | 15 | -12 | 45 | -4.236 | 0.000223 | 482 |
| 04_VAN_percent85_A10 | gradient3 | Cingulum_Mid_R | 15 | -12 | 45 | -4.236 | 0.000223 | 482 |
| 04_VAN_percent95_A00 | gradient1 | Insula_L | -24 | 15 | -18 | 4.650 | 0.000072 | 257 |
|  | gradient2 | SupraMarginal_R | 66 | -18 | 36 | 2.999 | 0.005631 | 286 |
|  | gradient3 | Cingulum_Mid_R | 9 | -36 | 36 | 4.375 | 0.000153 | 205 |
| 04_VAN_percent95_A05 | gradient1 | Insula_L | -24 | 15 | -18 | 4.650 | 0.000072 | 257 |
|  | gradient2 | SupraMarginal_R | 66 | -18 | 36 | 2.999 | 0.005631 | 286 |
|  | gradient3 | Cingulum_Mid_R | 9 | -36 | 36 | 4.375 | 0.000153 | 205 |
| 04_VAN_percent95_A10 | gradient1 | Insula_L | -24 | 15 | -18 | 4.650 | 0.000072 | 257 |
|  | gradient2 | SupraMarginal_R | 66 | -18 | 36 | 2.999 | 0.005631 | 286 |
|  | gradient3 | Cingulum_Mid_R | 9 | -36 | 36 | 4.375 | 0.000153 | 205 |
| 05_LIM_percent85_A00 | gradient2 | Temporal_Pole_Mid_R | 48 | 9 | -27 | 4.891 | 0.000037 | 291 |
|  | gradient3 | Frontal_Sup_Orb_R | 15 | 18 | -21 | -6.658 | 0.000000 | 129 |
| 05_LIM_percent85_A05 | gradient2 | Temporal_Pole_Mid_R | 48 | 9 | -27 | 4.891 | 0.000037 | 291 |
|  | gradient3 | Frontal_Sup_Orb_R | 15 | 18 | -21 | -6.658 | 0.000000 | 129 |
| 05_LIM_percent85_A10 | gradient2 | Temporal_Pole_Mid_R | 48 | 9 | -27 | 4.891 | 0.000037 | 291 |
|  | gradient3 | Frontal_Sup_Orb_R | 15 | 18 | -21 | -6.658 | 0.000000 | 129 |
| 05_LIM_percent95_A00 | gradient2 | Temporal_Pole_Mid_R | 36 | 6 | -30 | 3.847 | 0.000632 | 308 |
| 05_LIM_percent95_A05 | gradient2 | Temporal_Pole_Mid_R | 36 | 6 | -30 | 3.847 | 0.000632 | 308 |
| 05_LIM_percent95_A10 | gradient2 | Temporal_Pole_Mid_R | 36 | 6 | -30 | 3.847 | 0.000632 | 308 |
| 06_FPN_percent85_A00 | gradient3 | Frontal_Inf_Tri_L | -51 | 27 | 24 | -3.870 | 0.000595 | 185 |
| 06_FPN_percent85_A05 | gradient3 | Frontal_Inf_Tri_L | -51 | 27 | 24 | -3.870 | 0.000595 | 185 |
| 06_FPN_percent85_A10 | gradient3 | Frontal_Inf_Tri_L | -51 | 27 | 24 | -3.870 | 0.000595 | 185 |
| 07_DMN_percent80_A00 | gradient2 | Precuneus_L | -15 | -66 | 60 | -3.488 | 0.001626 | 337 |
|  |  | Angular_L | -51 | -60 | 36 | 3.148 | 0.003883 | 179 |
|  | gradient3 | Precuneus_L | -12 | -63 | 66 | 5.073 | 0.000023 | 376 |
|  |  | Cingulum_Ant_L | -9 | 39 | 15 | -3.706 | 0.000919 | 362 |
| 07_DMN_percent80_A05 | gradient2 | Precuneus_L | -15 | -66 | 60 | -3.488 | 0.001626 | 337 |
|  |  | Angular_L | -51 | -60 | 36 | 3.148 | 0.003883 | 179 |
|  | gradient3 | Precuneus_L | -12 | -63 | 66 | 5.073 | 0.000023 | 376 |
|  |  | Cingulum_Ant_L | -9 | 39 | 15 | -3.706 | 0.000919 | 362 |
| 07_DMN_percent80_A10 | gradient2 | Precuneus_L | -15 | -66 | 60 | -3.488 | 0.001626 | 337 |
|  |  | Angular_L | -51 | -60 | 36 | 3.148 | 0.003883 | 179 |
|  | gradient3 | Precuneus_L | -12 | -63 | 66 | 5.073 | 0.000023 | 376 |
|  |  | Cingulum_Ant_L | -9 | 39 | 15 | -3.706 | 0.000919 | 362 |
| 07_DMN_percent85_A00 | gradient2 | Temporal_Mid_L | -60 | -3 | -12 | 4.784 | 0.000050 | 743 |
|  |  | Temporal_Mid_R | 63 | -42 | 12 | 4.464 | 0.000120 | 715 |
|  |  | Frontal_Sup_R | 21 | 63 | 12 | -5.820 | 0.000003 | 634 |
|  | gradient3 | Frontal_Sup_Medial_R | 3 | 21 | 45 | -4.911 | 0.000035 | 278 |
| 07_DMN_percent85_A05 | gradient2 | Temporal_Mid_L | -60 | -3 | -12 | 4.784 | 0.000050 | 743 |
|  |  | Temporal_Mid_R | 63 | -42 | 12 | 4.464 | 0.000120 | 715 |
|  |  | Frontal_Sup_R | 21 | 63 | 12 | -5.820 | 0.000003 | 634 |
|  | gradient3 | Frontal_Sup_Medial_R | 3 | 21 | 45 | -4.911 | 0.000035 | 278 |
| 07_DMN_percent85_A10 | gradient2 | Temporal_Mid_L | -60 | -3 | -12 | 4.784 | 0.000050 | 743 |
|  |  | Temporal_Mid_R | 63 | -42 | 12 | 4.464 | 0.000120 | 715 |
|  |  | Frontal_Sup_R | 21 | 63 | 12 | -5.820 | 0.000003 | 634 |
|  | gradient3 | Frontal_Sup_Medial_R | 3 | 21 | 45 | -4.911 | 0.000035 | 278 |
| 07_DMN_percent95_A00 | gradient2 | Cingulum_Ant_R | 6 | 36 | 15 | -4.109 | 0.000314 | 717 |
|  | gradient3 | Cingulum_Ant_L | -6 | 36 | 30 | -3.951 | 0.000479 | 613 |
|  |  | Precuneus_L | -15 | -48 | 60 | 3.856 | 0.000617 | 407 |
| 07_DMN_percent95_A05 | gradient2 | Cingulum_Ant_R | 6 | 36 | 15 | -4.109 | 0.000314 | 717 |
|  | gradient3 | Cingulum_Ant_L | -6 | 36 | 30 | -3.951 | 0.000479 | 613 |
|  |  | Precuneus_L | -15 | -48 | 60 | 3.856 | 0.000617 | 407 |
| 07_DMN_percent95_A10 | gradient2 | Cingulum_Ant_R | 6 | 36 | 15 | -4.109 | 0.000314 | 717 |
|  | gradient3 | Cingulum_Ant_L | -6 | 36 | 30 | -3.951 | 0.000479 | 613 |
|  |  | Precuneus_L | -15 | -48 | 60 | 3.856 | 0.000617 | 407 |

percent80 percent85 percent95 denotes that the connection matrix threshold is the first 20% 15% , 5%;A00, A05,A10 denotes that α is 0, 0.5, 1 respectively

Male Gradient Calculations

Sensitivity analyses were performed using different connection thresholds (e.g., 5%, 15%, 20%) and alpha values (e.g., 0, 0.5, 1) to demonstrate the stability of the resulting gradients;

Threshold combinations 5%-0 5%-0.5 5%-1

15%-0 15%-0.5 15%-1

20%-0 20%-0.5 20%-1

Table S9 Functional gradients of seven brain networks in males under multiple parameter settings

| 参数 | 梯度 | 脑区 | MNI峰值坐标 | | | T value | P value | Cluster Size |
| --- | --- | --- | --- | --- | --- | --- | --- | --- |
|  |  |  | X | Y | Z |  |  |  |
| 02_SMN_percent80_A00 | gradient3 | Rolandic_Oper_R | 48 | -21 | 21 | -3.250 | 0.001818 | 243 |
| 02_SMN_percent80_A05 | gradient3 | Rolandic_Oper_R | 48 | -21 | 21 | -3.250 | 0.001818 | 243 |
| 02_SMN_percent80_A10 | gradient3 | Rolandic_Oper_R | 48 | -21 | 21 | -3.250 | 0.001818 | 243 |
| 02_SMN_percent85_A00 | gradient2 | Postcentral_R | 15 | -48 | 72 | -3.650 | 0.000519 | 619 |
| 02_SMN_percent85_A05 | gradient2 | Postcentral_R | 15 | -48 | 72 | -3.650 | 0.000519 | 619 |
| 02_SMN_percent95_A00 | gradient2 | Precentral_R | 45 | -6 | 36 | 3.113 | 0.002738 | 343 |
|  | gradient3 | Precentral_R | 54 | 0 | 21 | -4.460 | 0.000033 | 412 |
|  |  | Postcentral_L | -21 | -27 | 66 | -3.407 | 0.001123 | 339 |
|  |  | Postcentral_L | -57 | -18 | 18 | -3.530 | 0.000763 | 280 |
| 02_SMN_percent95_A05 | gradient3 | Precentral_R | 54 | 0 | 21 | -4.460 | 0.000033 | 412 |
|  |  | Postcentral_L | -21 | -27 | 66 | -3.407 | 0.001123 | 339 |
|  |  | Postcentral_L | -57 | -18 | 18 | -3.530 | 0.000763 | 280 |
| 02_SMN_percent95_A10 | gradient3 | Precentral_R | 54 | 0 | 21 | -4.460 | 0.000033 | 412 |
|  |  | Postcentral_L | -21 | -27 | 66 | -3.407 | 0.001123 | 339 |
|  |  | Postcentral_L | -57 | -18 | 18 | -3.530 | 0.000763 | 280 |
| 03_DAN_percent85_A00 | gradient2 | Temporal_Inf_R | 57 | -9 | -39 | -3.219 | 0.001996 | 200 |
| 03_DAN_percent85_A05 | gradient2 | Temporal_Inf_R | 57 | -9 | -39 | -3.219 | 0.001996 | 200 |
| 03_DAN_percent85_A10 | gradient2 | Temporal_Inf_R | 57 | -9 | -39 | -3.219 | 0.001996 | 200 |
| 04_VAN_percent85_A00 | gradient1 | Insula_L | -39 | 21 | 0 | 3.008 | 0.003719 | 178 |
| 04_VAN_percent85_A05 | gradient1 | Insula_L | -39 | 21 | 0 | 3.008 | 0.003719 | 178 |
| 04_VAN_percent85_A10 | gradient1 | Insula_L | -39 | 21 | 0 | 3.008 | 0.003719 | 178 |
| 04_VAN_percent95_A00 | gradient3 | Insula_R | 33 | 27 | 6 | 3.329 | 0.001429 | 175 |
| 04_VAN_percent95_A05 | gradient3 | Insula_R | 33 | 27 | 6 | 3.329 | 0.001429 | 175 |
| 04_VAN_percent95_A10 | gradient3 | Insula_R | 33 | 27 | 6 | 3.329 | 0.001429 | 175 |
| 07_DMN_percent80_A00 | gradient2 | Temporal_Mid_L | -51 | -63 | 12 | -3.430 | 0.001045 | 276 |
|  | gradient3 | Frontal_Med_Orb_R | 9 | 60 | -15 | 4.141 | 0.000100 | 380 |
| 07_DMN_percent80_A05 | gradient2 | Temporal_Mid_L | -51 | -63 | 12 | -3.430 | 0.001045 | 276 |
|  | gradient3 | Frontal_Med_Orb_R | 9 | 60 | -15 | 4.569 | 0.000022 | 538 |
| 07_DMN_percent80_A10 | gradient2 | Temporal_Mid_L | -51 | -63 | 12 | -3.430 | 0.001045 | 276 |
|  | gradient3 | Frontal_Med_Orb_R | 9 | 60 | -15 | 4.141 | 0.000100 | 380 |
| 07_DMN_percent85_A00 | gradient1 | Temporal_Mid_L | -54 | -42 | 6 | -3.208 | 0.002063 | 327 |
|  | gradient3 | Cingulum_Post_L | -9 | -45 | 27 | 3.539 | 0.000741 | 384 |
| 07_DMN_percent85_A05 | gradient1 | Temporal_Mid_L | -54 | -42 | 6 | -3.208 | 0.002063 | 327 |
|  | gradient3 | Cingulum_Post_L | -9 | -45 | 27 | 3.539 | 0.000741 | 384 |
| 07_DMN_percent85_A10 | gradient1 | Temporal_Mid_L | -54 | -42 | 6 | -3.208 | 0.002063 | 327 |
|  | gradient3 | Cingulum_Post_L | -9 | -45 | 27 | 3.539 | 0.000741 | 384 |
| 07_DMN_percent95_A00 | gradient2 | Temporal_Mid_R | 66 | -48 | 12 | 4.002 | 0.000162 | 311 |
|  |  | Temporal_Mid_L | -45 | -69 | 12 | 4.191 | 0.000084 | 203 |
| 07_DMN_percent95_A05 | gradient2 | Temporal_Mid_R | 66 | -48 | 12 | 4.002 | 0.000162 | 311 |
|  |  | Temporal_Mid_L | -45 | -69 | 12 | 4.191 | 0.000084 | 203 |
| 07_DMN_percent95_A10 | gradient2 | Temporal_Mid_R | 66 | -48 | 12 | 4.002 | 0.000162 | 311 |
|  |  | Temporal_Mid_L | -45 | -69 | 12 | 4.191 | 0.000084 | 203 |

percent80 percent85 percent95 denotes that the connection matrix threshold is the first 20% 15% , 5%;A00, A05,A10 denotes that α is 0, 0.5, 1 respectively

Result:The analysis indicates that, regardless of the threshold or α value, the brain regions showing between-group differences in gradients 1–3 remain highly consistent, demonstrating the robustness of our conclusions to parameter choices.
